# Dynamic Evolution of Volatile Aroma Compounds in Black Ginseng During Nine-Steaming and Nine-Drying Based on HS-GC-IMS and Chemometrics

**DOI:** 10.3390/foods15173031

**Published:** 2026-08-27

**Authors:** Hui Zhao, Rui Wang, Zhiwei Feng, Tianxing Zhao, Hongying Guo, Yuhe Ren, Youde Ma, Rongbing Bi, Meiling Jin, Lili Cui

**Affiliations:** 1 Institute of Special Wild Economic Animal and Plant Science, Chinese Academy of Agricultural Sciences, Changchun 130112, China; zhaohui02@caas.cn (H.Z.); 18744108018@163.com (R.W.); zhaotx_1998@163.com (T.Z.); gerhyy@163.com (H.G.); renyuhe1123@163.com (Y.R.); birongbing@caas.cn (R.B.); lovemeiling521@hotmail.com (M.J.); 2 Jilin Shenwang Plant Protection Co., Ltd., Baishan 134500, China; cbsfeng@126.com (Z.F.); cbsma@126.com (Y.M.)

**Keywords:** volatile aroma compounds, HS-GC-IMS, PLS-DA, ROAV, Maillard reaction

## Abstract

Headspace–gas chromatography–ion mobility spectrometry (HS-GC-IMS) combined with chemometrics was employed to track volatile aroma compounds during the nine-steaming and nine-drying process. A total of 84 volatile signals (including monomers, dimers, and polymers) were tentatively identified, of which 73 volatile compounds were semi-quantified. Principal component analysis (PCA) revealed a mixed clustering pattern across cycles, indicating that unsupervised methods alone could not adequately resolve the volatile changes. A partial least squares discriminant analysis (PLS-DA) model supported clear chemical discrimination among the three hypothesized stages—early (cycles 1–3), middle (4–6), and late (7–9). Based on VIP > 1 and ANOVA (*q* < 0.05), 29 key differential compounds were screened and categorized into three dynamic groups (decreasing, transient, and accumulating), revealing three proposed transformation windows: green-grassy odor dissipation (cycles 1–3), roasted/malty aroma generation (cycles 4–6), and aroma stabilization via end-product accumulation (cycles 7–9). Relative odor activity value (ROAV) analysis revealed a decoupling between abundance and sensory impact: high-abundance Maillard products like maltol showed negligible ROAV (<0.0003), whereas esters and aldehydes dominated the ROAV profiles, suggesting their potential as important contributors to the fruity, sweet, and malty notes. The maltol/α-cedrol ratio (M/C) increased by an order of magnitude after the seventh steaming, and color parameters E*ab correlated negatively with ester accumulation. This study provides chemical insights and quantitative indicators for aroma quality control and process determination in black ginseng production.

## 1. Introduction

*Panax ginseng* C.A. Mey., a perennial herbaceous plant belonging to the Araliaceae family, is reputed as the “King of Herbs” and has been traditionally used as a tonic in Asian countries such as China, Korea, and Japan [1]. Black ginseng is a processed product made from fresh ginseng through repeated processing, and it exhibits significant biological activities, including antioxidant, antitumor, anti-inflammatory, and immunomodulatory effects [2,3,4].

Additionally to bioactivities, aroma quality is also an important indicator for evaluating the processing quality of black ginseng. During the nine-steaming and nine-drying process, the repeated moist-heat treatment not only promotes the transformation of saponins but is also accompanied by complex chemical processes such as polysaccharide degradation, amino acid release, lipid oxidation, and the Maillard reaction, thereby forming the characteristic caramelized sweet, roasted, and fruity aroma of black ginseng [5,6,7,8].

Previous studies have shown that volatile components such as terpenoids, aldehydes, furans, and nitrogen-containing heterocyclic compounds play important roles in the formation of black ginseng aroma, and their compositional and content variations are closely related to processing conditions [9]. In recent years, significant progress has been made in research on volatile components of Panax species: a study using GC-IMS to compare the effects of five drying methods on the VOCs of fresh ginseng tentatively identified 68 compounds and screened 29 characteristic markers [10]. Another study on different Panax species utilized GC-IMS to distinguish among red ginseng, American ginseng, and Asian ginseng, revealing that sesquiterpenoids are the characteristic volatile substances of the three and that heating can promote their interconversion [11]. It was also found that the caramelized and medicinal aroma of black ginseng is closely associated with the accumulation of high levels of furans, pyran esters, and aromatic compounds [12]. Most existing studies are based on static comparisons among different final processed products, and systematic understanding of the dynamic evolution of volatile components during the nine-steaming and nine-drying process remains lacking.

The repeated steaming and drying treatments during the nine-steaming and nine-drying process may induce complex continuous transformations of volatile components, and their variation pattern is not a simple linear one but may involve stage-specific transitions and critical turning points. Such dynamic characteristics have been reported in other traditional medicinal materials subjected to nine-steaming and nine-drying. During the processing of Polygonatum (a non-Panax medicinal plant), aldehydes decreased significantly, whereas acids and ketones gradually increased [13]; in the case of nine-steaming and nine-drying black sesame, most quality indicators tended to stabilize after a certain number of steaming and drying cycles [14]. Existing studies suggest that traditional steaming and drying processes are driven by the interplay of multiple reactions and exhibit stage-specific evolution characteristics. It remains to be investigated whether similar critical transition windows exist during the aroma formation of black ginseng and whether statistically significant chemical markers truly determine the sensory aroma quality. This knowledge gap limits the establishment of quality control standards and the optimization of processing technology for black ginseng.

Headspace–gas chromatography–ion mobility spectrometry (HS-GC-IMS) combines the high separation capability of gas chromatography with the high sensitivity of ion mobility spectrometry, enabling rapid and high-throughput detection of volatile components without complex sample pretreatment, and has been widely applied in the analysis of volatile components during the processing of food and Chinese medicinal materials [15]. When combined with chemometric methods such as principal component analysis (PCA), partial least squares discriminant analysis (PLS-DA), and hierarchical cluster analysis (HCA), it can effectively resolve key differential information in complex samples [16].

Therefore, this study employed HS-GC-IMS combined with chemometric methods to dynamically track the volatile aroma compounds throughout the entire nine-steaming and nine-drying process (the first to the ninth steaming and drying cycles) of black ginseng, aiming to (1) elucidate the overall evolution patterns and stage characteristics of volatile components during the process; (2) screen key differential aroma markers and identify candidate transition windows of aroma changes; (3) evaluate the theoretical aroma contribution of key compounds based on relative odor activity value (ROAV) and explore the relationship between chemical markers and estimated sensory contributions; and (4) propose convenient empirical process endpoint indicators, thereby providing a theoretical basis for the quality control and optimization of black ginseng processing.

## 2. Materials and Methods

### 2.1. Samples and Reagents

Five-year-old fresh ginseng (*Panax ginseng* C.A. Mey.) with an average single root weight of 105.3 ± 5.2 g (mean ± SD, *n* = 27) was collected from a ginseng base in Fusong County, Changbai Mountain Protection Development Zone, Jilin Province, China. A total of 27 fresh ginseng roots were randomly divided into 3 independent processing batches (9 roots per batch). Referring to DB21/T 3379-2021 Technical Regulation for Production of Black Ginseng [17], the traditional nine-steaming and nine-drying process was adopted: For the first and second steaming cycles, the temperature was gradually increased from 40 °C to 90 °C, with a 30 min holding time at each 10 °C increment; upon reaching 90 °C, steaming was continued for 6 h. From the third to the ninth steaming cycles, each cycle lasted 8 h at a controlled temperature of 90 ± 5 °C. After each steaming, the samples were dried for 6 h at a controlled temperature of 55 ± 5 °C. The samples were ground, passed through a 60-mesh sieve, sealed, and stored at −20 °C. For each batch, the nine-steaming and nine-drying process was carried out continuously for 9 cycles. At the end of each complete steaming–drying cycle, one root was randomly taken from each of the 3 batches, individually ground, and analyzed. After the nine-steaming and nine-drying process, the black ginseng samples had a moisture content of approximately 40–45%. For volatile compound analysis, a portion of each sample was further dried to a uniform moisture content of ~8%, and all concentrations are reported on a dry weight basis (mg/kg dry matter).

### 2.2. Colorimetric Measurement

A NH300 colorimeter (Shenzhen 3NH Technology Co., Ltd., Shenzhen, China) was used, referring to the method of Jiang Changpeng [18]. The measurement conditions were set as follows: D65 light source (natural daylight, color temperature 6504 K), reflectance mode, 10° observer angle, and 4 mm measurement aperture. The CIELAB color vector magnitude (referred to hereafter as E*ab) was calculated using the following formula: E*ab = [(L*)^2^ + (a*)^2^ + (b*)^2^]^1^/^2^. This parameter represents the distance of the measured color point from the origin of the CIELAB space. After black-and-white plate calibration, 1.00 g of sample powder was accurately weighed and spread evenly on the color measurement plate to form a flat surface. The plate was then properly installed to prevent the powder from shifting. The L, a, and b values were sequentially measured and recorded, and the CIELAB color vector magnitude E*ab was calculated. Each sample was measured in triplicate, and the average values were recorded. The unprocessed sample (cycle 0) was photographed and measured for L*, a*, and b* values but was excluded from the statistical analyses (ANOVA, correlation) which were based on the nine processed samples (cycles 1–9, *n* = 9).

### 2.3. HS-GC-IMS Analysis

HS-GC-IMS measurements were carried out on a FlavourSpec^®^ instrument (G.A.S. GmbH, Dortmund, Germany) fitted with an MXT-5 capillary column (15 m × 0.53 mm, 1.0 μm), according to the procedure of Yuzhang Mi [19]. Each sample (0.5000 g) was placed in a 20 mL headspace vial with 5 μL of internal standard (2500 μg/mL), and then incubated at 80 °C for 15 min while shaking at 500 rpm. A 500 μL aliquot of the headspace was injected using a syringe maintained at 85 °C, and the column oven was held at 60 °C. High-purity N_2_ served as the carrier gas, programmed as follows: 2 mL/min for 5 min; ramped to 10 mL/min over 3 min, to 50 mL/min over 3 min, to 100 mL/min over 10 min, and to 150 mL/min over 10 min; and finally held for 30 min. The drift tube was operated at 45 °C with a drift gas (N_2_) flow of 75 mL/min and an electric field of 500 V/cm. Compounds were tentatively identified by matching experimental retention indices (RIs) and relative drift times against two reference libraries. The NIST 2020 Retention Index Library was used to match RI values and propose candidate structures based on GC retention on equivalent stationary phases. The G.A.S. GC × IMS Library (v1.0.3) supplied reference drift times from standardized IMS spectra. An identification was considered acceptable when both the absolute RI deviation (<20) and the absolute drift-time difference (<0.01 ms) fell within these tolerances relative to the library data.

### 2.4. Semi-Quantification

Semi-quantification was carried out via the internal standard (IS) method, using the formula

$$W_{x}=\frac{C_{is}\;\times \;V_{is}}{1000\;\times \;m}\times \frac{A_{x}}{A_{is}}$$

where *W_x_* is the target compound content, *C_is_* the IS concentration (µg/mL), *V_is_* the IS volume added (µL), m the sample weight (g), and *A_x_* and *A_is_* are the peak areas of the target compound and IS, respectively.

GC-IMS calibration standards (C_4_–C_9_ n-ketones) and the IS (2-methyl-3-heptanone, 99.5% purity) were obtained from Aladdin (Shanghai, China) and Tan-Mo Technology (Changzhou, China), respectively. The IS was added at 25 mg/kg in each sample (5 µL of a 2500 µg/mL solution to 0.5000 g of powder), and all concentrations are given on a dry weight basis.

For quality control, each analytical batch contained a blank vial to monitor background and carryover. Instrument drift was assessed by injecting n-ketone standards at the start, middle, and end of each batch (RSD of IS peak volume < 8.5%). Method repeatability, determined by six consecutive injections of the same sample, gave RSDs of 3.2–9.7% for representative compounds.

In HS-GC-IMS, compounds with sufficient proton affinity may form both monomer (M) and dimer (D) ions, with their distribution governed by a concentration-dependent equilibrium: monomers predominate at low concentrations, while dimers become stronger at higher concentrations. For semi-quantification, a single ionic species was selected for each compound: the dimer was used when detectable; otherwise, the monomer was used.

### 2.5. Relative Odor Activity Value (ROAV) Analysis

To evaluate the contribution of each volatile component to the overall aroma of black ginseng, the relative odor activity value (ROAV) method was employed for analysis, with the following formula [20]:

$${ROAV}_{i}\;=\;\frac{C_{i}/T_{i}}{C_{max}/T_{max}}\times 10$$

Note: *C_i_* represents the content of compound *i* (mg/kg, semi-quantified using an internal standard); *T_i_* represents the aroma threshold of compound *i* in water (mg/kg, obtained by consulting the literature and databases). The compound exhibiting the highest (C/T) value in each steaming and drying cycle was assigned an ROAV of 100. ROAV ≥ 1 indicates that the compound is a key aroma contributor. Aroma thresholds were retrieved from the Leibniz-LSB@TUM Odorant Database (https://www.leibniz-lsb.de), with some supplemented from literature reports. All odor thresholds used in this study were determined in water. Therefore, the ROAVs represent theoretical estimates in an aqueous matrix.

### 2.6. Data Processing and Statistical Analysis

All experiments were performed in triplicate, and data are expressed as mean ± standard deviation (SD). GC-IMS data were processed with VOCal software (version 0.4.03, G.A.S. GmbH). For multivariate analyses, the data were mean-centered and scaled to unit variance. Normality of residuals was assessed using the Shapiro–Wilk test, and homogeneity of variances was assessed using Levene’s test. When these assumptions were violated, the Kruskal–Wallis test was used as a non-parametric alternative. Principal component analysis (PCA), partial least squares discriminant analysis (PLS-DA), and variable importance in projection (VIP) scores were obtained using SIMCA 14.1 (Umetrics, Malmö, Sweden); model robustness was evaluated by 7-fold cross-validation, and model significance was assessed using CV-ANOVA. SPSS 26.0 was used for partial correlation analysis, Spearman’s rank correlation analysis. Pairwise comparisons were adjusted for multiple testing using the Benjamini–Hochberg FDR method (*q* < 0.05). Heatmaps, scatter plots, aroma wheel charts, stacked bar charts, and radar charts were generated with Origin 2024 Pro and RAWGraphs 2.0, while heatmap were specifically prepared using Origin 2024 Pro.

## 3. Results

### 3.1. Identification of Volatile Components by HS-GC-IMS

**Figure 1:**
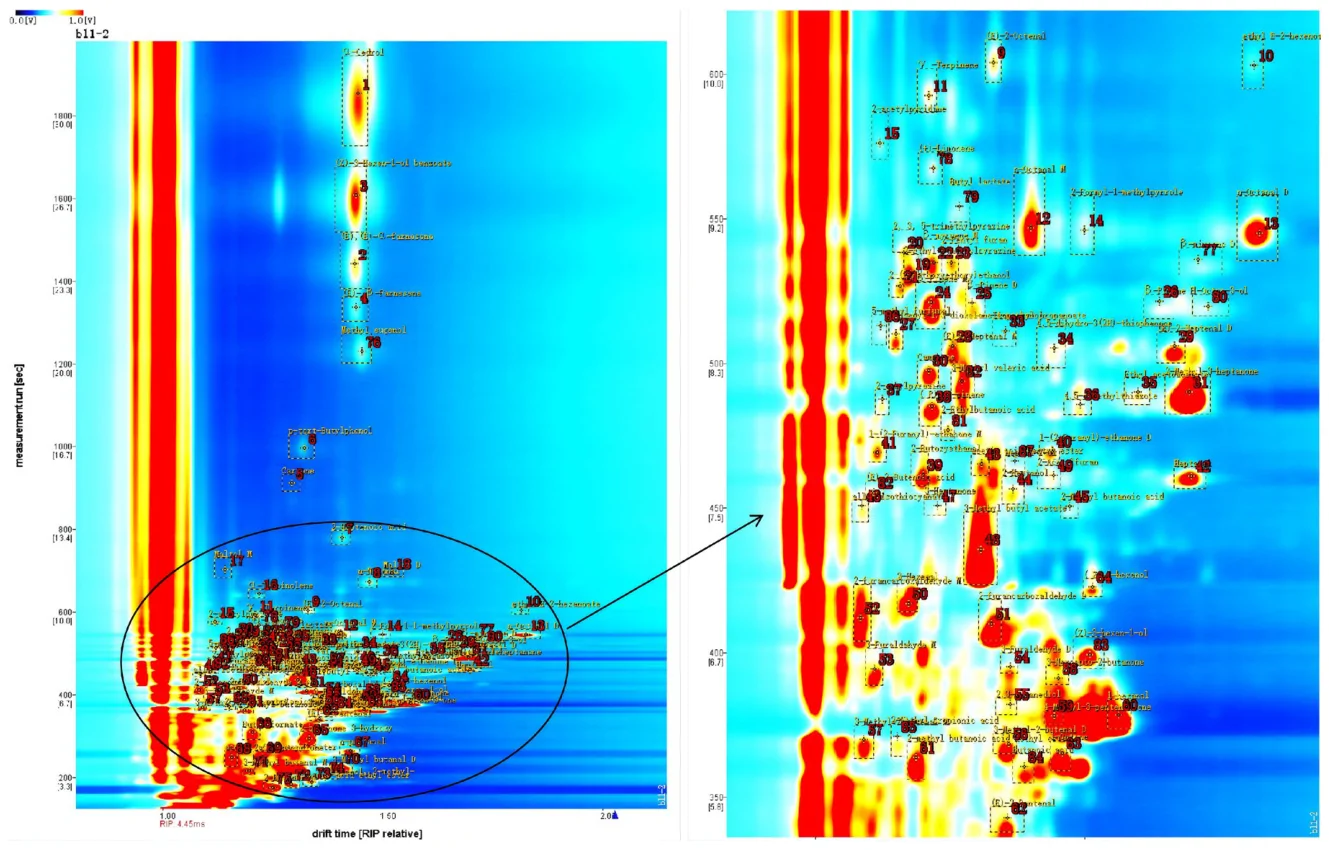
HS-GC-IMS two-dimensional topographic plot of volatile components in the nine-steaming and nine-drying process of black ginseng.

The black ginseng samples from the nine-steaming and nine-drying process were analyzed using HS-GC-IMS technology. The typical two-dimensional spectrum is presented in Figure 1. In the spectrum, the vertical axis represents the gas chromatographic retention time, and the horizontal axis represents the relative drift time of ions. The vertical red line on the left side of the spectrum is the reaction ion peak (RIP), which is formed by the reactant ions generated by the tritium ionization source and serves as an internal reference signal for drift time calibration and spectrum alignment. Each signal spot to the right of the RIP corresponds to a volatile component, and the spot color is positively correlated with the signal intensity of the component. The closer the color is to bright red, the higher the signal intensity and relative abundance of the corresponding component; the lighter the color, the lower the relative abundance.

**Figure 2:**
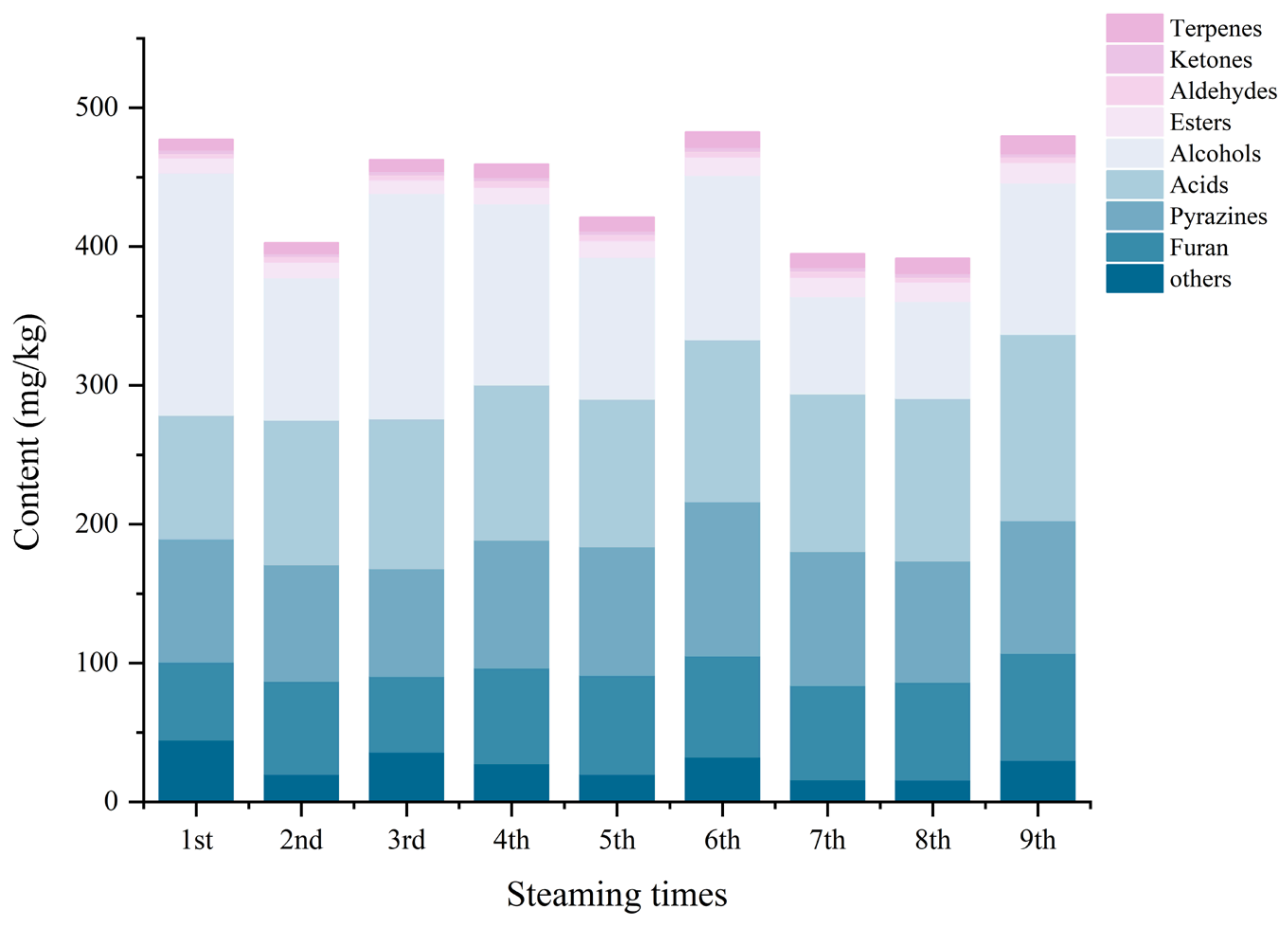
Classification and quantitative distribution of tentatively identified volatile components in the nine-steaming and nine-drying process of black ginseng by chemical categories.

Through database matching and qualitative identification, a total of 84 volatile signals were tentatively identified, which reduced to 73 volatile components after merging monomer and dimer forms. Based on their chemical structures, these components could be classified into 10 categories: terpenes (11), aldehydes (16), ketones (9), esters (11), alcohols (8), acids (7), pyrazines (3), furans (2), phenols (1), and others (5) (Figure 2). Detailed information, including the retention time, drift time, and relative abundance of all tentatively identified compounds, is provided in Supplementary Table S1.

**Figure 3:**
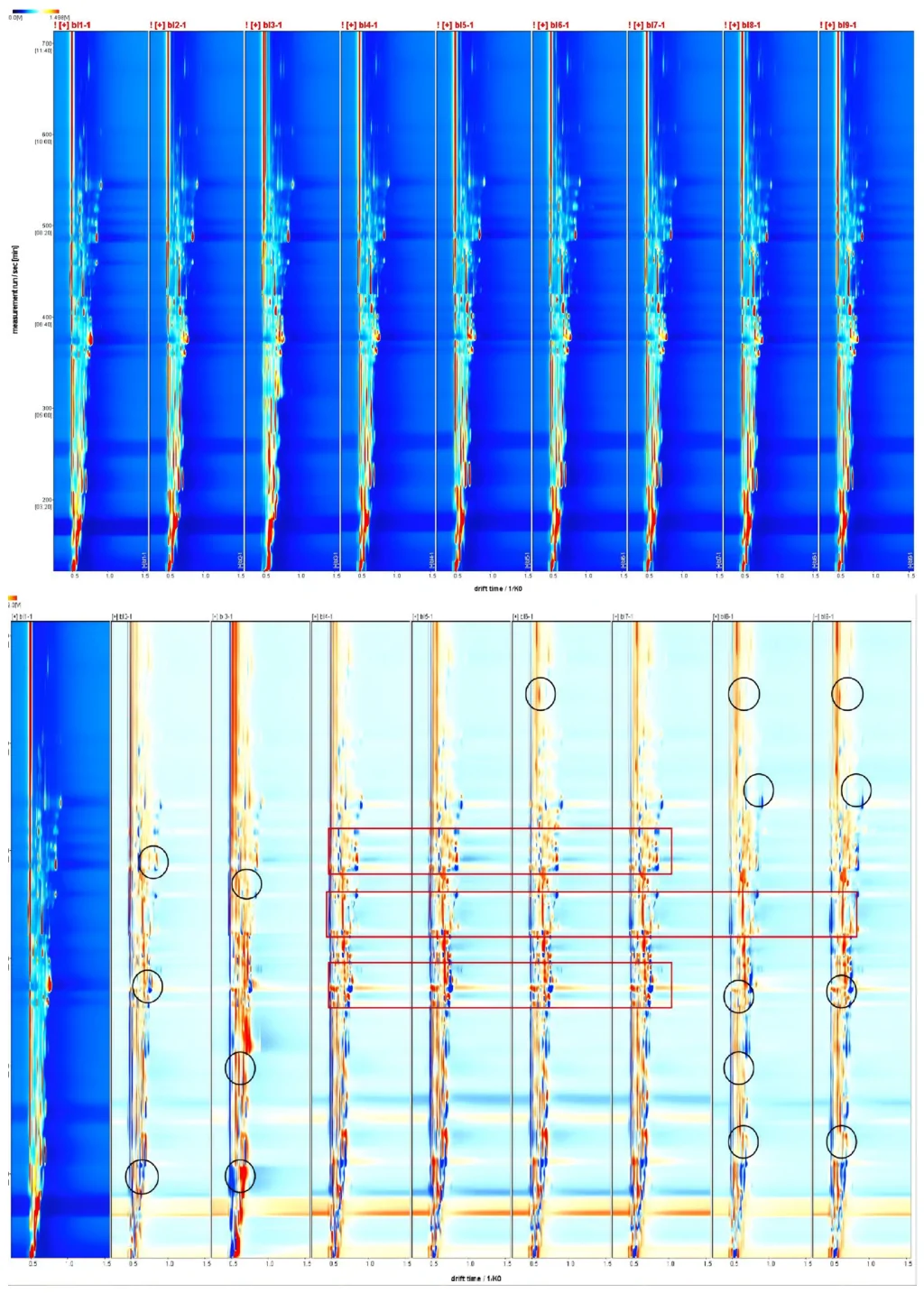
Two-dimensional (**top**) and comparative fingerprint map (**below**) of flavor compounds in black ginseng.

**Figure 4:**
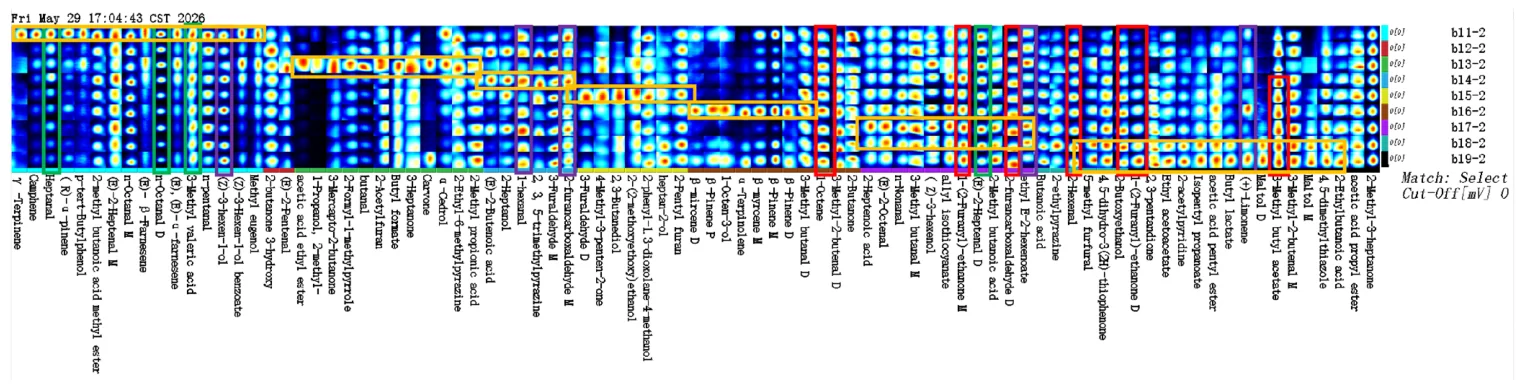
Fingerprint profiles of black ginseng powder samples across steaming cycles.

The two-dimensional and differential GC-IMS spectra and fingerprint maps (Figure 3 and Figure 4) showed that, with increasing steaming and drying cycles, the dynamic changes in volatile component abundance exhibited clear stage-specific patterns, which highly corresponded to the chemical reaction pathways during processing. In the early stage of steaming, natural terpenes (e.g., camphene, β-myrcene, α-cedrol) and primary lipid oxidation products (e.g., n-octanal, butanal, and other straight-chain aliphatic aldehydes) were predominant; in the middle and late stages, compounds potentially derived from Maillard reaction and Strecker degradation products (e.g., characteristic aldehydes such as 3-methylbutanal and 5-methylfurfural, ketones such as 2,3-pentanedione, and branched-chain fatty acids such as 2-ethylbutanoic acid) along with esterification products (e.g., isopentyl propanoate and ethyl acetoacetate) became dominant [21].

### 3.2. Global Nonlinear Fluctuations and Core Stage Signals

Based on precedents in the literature in similar nine-steaming and nine-drying processes [14,22,23,24,25], we hypothesized that the nine steaming–drying cycles might exhibit three distinct phases—early (cycles 1–3), middle (cycles 4–6), and late (cycles 7–9). The unsupervised PCA showed a mixed clustering pattern, indicating that the volatile changes were nonlinear and could not be reliably grouped by unsupervised methods alone. The PCA score plot showed that the first two principal components explained 39.5% and 13.1% of the total variance, respectively, accounting for 52.6% cumulatively. The cumulative R^2^X of the full PCA model with all components was 0.984.

**Figure 5:**
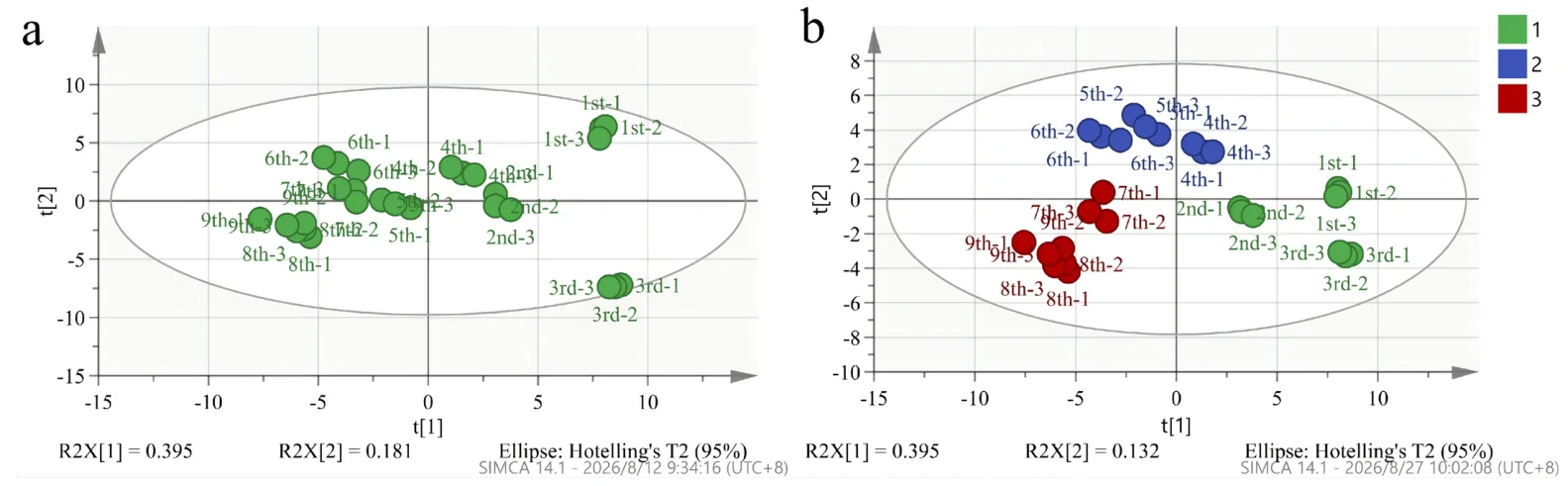
Unsupervised and supervised multivariate analysis of volatile components in black ginseng during the nine-cycle steaming and drying process. (**a**) PCA and (**b**) PLS-DA score plots.

**Figure 6:**
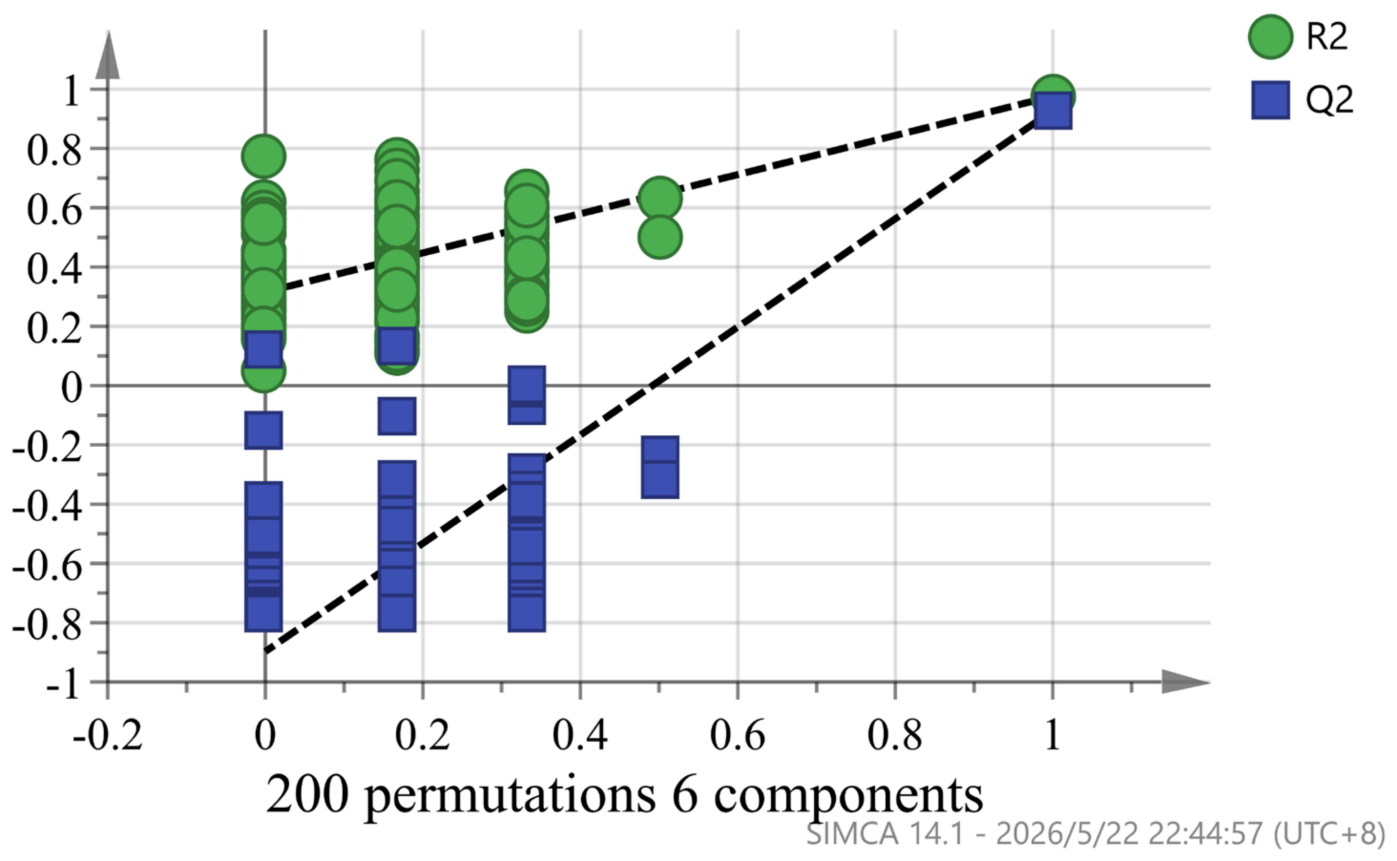
Permutation test for the PLS-DA model (200 iterations).

A PLS-DA model was constructed with cycles 1–3 (early stage), cycles 4–6 (middle stage), and cycles 7–9 (late stage) as Y variables (Figure 5b). The sample data corresponding to the three stages each clustered into distinct groups with clear boundaries. The model yielded R^2^Y = 0.977 and Q^2^ = 0.939. Data were preprocessed using unit variance (UV) scaling. The optimal number of latent variables (3) was determined by 7-fold cross-validation. CV-ANOVA indicated the model significance (*p* < 0.001). A 200-iteration permutation test (Figure 6) produced a Q^2^ intercept of −0.897, suggesting that the stage signals were genuine and not substantially influenced by noise [26]—substantial noise irrelevant to the grouping did not interfere with the model. Furthermore, CV-ANOVA yielded an F-value of 12.98 and a *p*-value of 6.01 × 10^−10^, demonstrating that the PLS-DA model is statistically highly significant. The confusion matrix indicated that all samples were correctly classified into their respective stages. However, it should be noted that the model’s performance was evaluated using random 7-fold cross-validation on the present dataset; its external generalizability to independent processing batches from different production years or regions remains to be tested.

### 3.3. Elucidation of the Aroma Formation Process

#### 3.3.1. Screening of Key Differential Markers and Positioning in the Reaction

To reliably identify volatile markers that distinguish different steaming stages, a dual-criteria screening strategy was employed. First, the variable importance in projection (VIP) values derived from the PLS-DA model were used to quantify the contribution of each compound to the inter-group discrimination. Second, the FDR-adjusted q-values from the one-way ANOVA (comparing the nine individual cycles) were used to confirm statistical significance.

**Figure 7:**
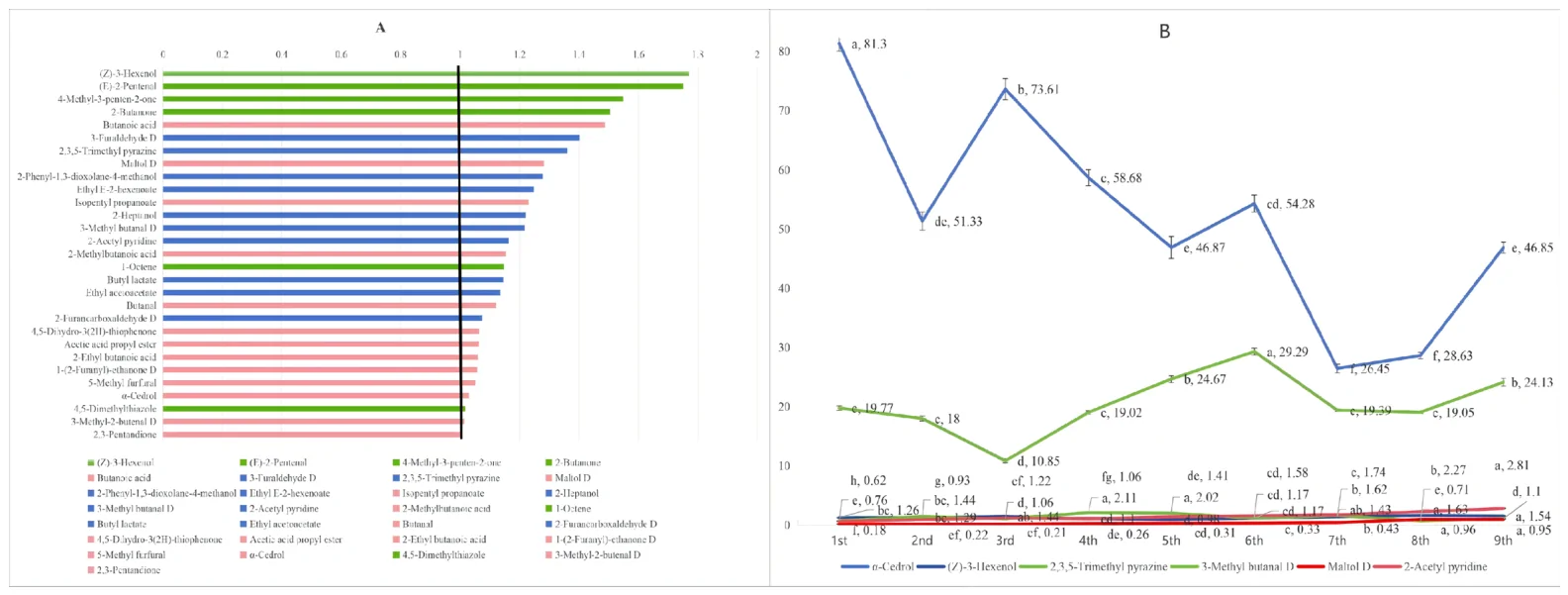
Thirty key differential volatile markers tentatively identified by the dual screening strategy (VIP > 1 and ANOVA *q* < 0.05). Note: (**A**) VIP scores from the PLS-DA model, colored by pattern-based groups (green: Group 1, decreasing; blue: Group 2, transient; pink: Group 3, accumulating). (**B**) Representative content variation trajectories (Mean ± SD, *n* = 3) for each role category. Different letters indicate significant differences among steaming cycles (*p* < 0.05).

Using PLS-DA VIP > 1 and ANOVA with FDR correction (*q* < 0.05) as the combined threshold, a total of 29 key differential aroma markers were tentatively identified (Figure 7A; complete data are provided in Table S2) [27]. Among them, (Z)-3-hexenol (VIP = 1.770) and (E)-2-pentenal (VIP = 1.751) exhibited the highest contributions, and the differences in content of all markers across different steaming and drying cycles were statistically significant (*p* < 0.05).

The 29 key differential markers were classified into three pattern-based groups based on their dynamic content variation patterns across the nine steaming and drying cycles (Table 1, Figure 7B):

(1) Group 1—Compounds with a decreasing trend (*n* = 6): These components exhibited relatively high contents during the initial steaming and drying stage and displayed an overall decreasing trend with increasing cycles, accompanied by slight fluctuations. Among them, α-cedrol exhibited the most pronounced decrease, with its content declining from 81.30 mg/kg to 6.45 mg/kg, corresponding to a total reduction of 92.1%, and showed a very strong negative correlation with the number of steaming and drying cycles (Spearman’s ρ = −0.91). Spearman’s rank correlation was used here because the relationships between compound contents and cycle number may be monotonic but not necessarily linear. These substances primarily contributed to the original green-grassy odor of ginseng, and their continuous decrease corresponded to the gradual dissipation of the green-grassy odor during processing.
(2) Group 2—Compounds with a transient/rise-and-fall trend (*n* = 9): These components exhibited multi-stage dynamic variation characteristics: their contents continuously decreased during the first to third steaming drying cycles, reached peak levels during the fourth to sixth cycles, declined during the seventh to eighth cycles, and increased again at the ninth cycle. 2,3,5-Trimethylpyrazine, 3-methylbutanal D, and 3-furaldehyde D were typical representatives. The Spearman correlation coefficients between these compounds and the number of steaming and drying cycles did not reach a significant level, indicating that their content changes did not follow a unidirectional linear trend but rather reflected their transient nature during processing.
(3) Group 3—Compounds with an accumulating trend (*n* = 14): These components exhibited a continuous accumulation trend with increasing steaming and drying cycles, and their contents tended to stabilize from the seventh to the ninth cycles. Among them, maltol D (increased 5.3-fold, ρ = 0.95) and 2-acetylpyridine (increased 4.5-fold, ρ = 0.93) were typical markers of Maillard reaction end-products [28]. Esters and short-chain fatty acid components also exhibited a unidirectional accumulation characteristic (ρ = 0.78–0.90).

#### 3.3.2. Flavor Transformation Window Periods

**Figure 8:**
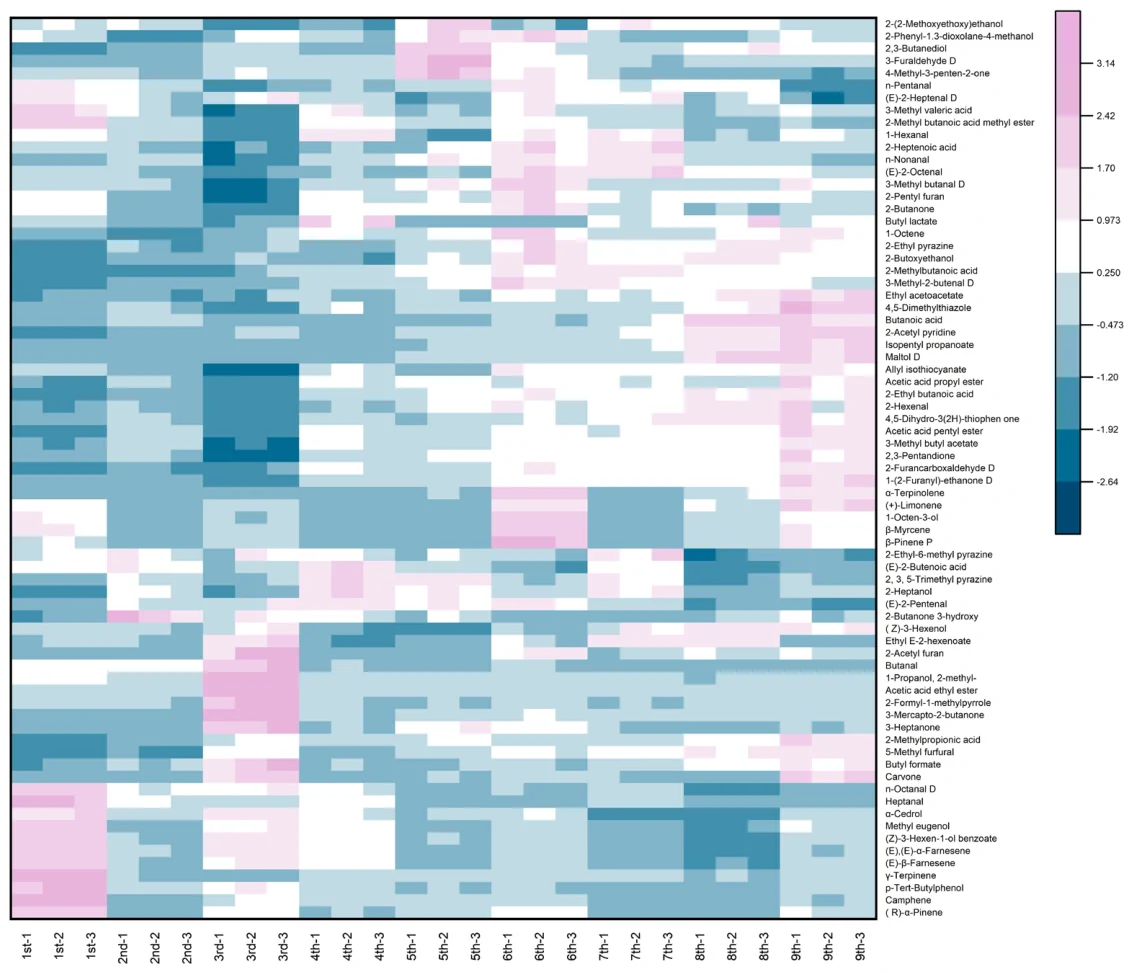
Heatmap of content variations in the 73 tentatively identified volatile compounds across nine steaming cycles. Note: Colors from green to pink represent Z-score-normalized contents (blue-green indicates low abundance and pink indicates high abundance).

Based on the normalized relative abundances of the 29 key differential markers, a hierarchical clustering heatmap was constructed (Figure 8) to verify the correspondence between component classification and processing stages. In the heatmap, rows represent the first to ninth steaming and drying samples for ginseng, columns represent volatile compounds, and colors ranging from dark green to pink correspond to normalized relative abundances from low to high.

The results showed that the 73 differential markers could be grouped into three characteristic clusters, and the nine samples with different processing degrees could be divided into three consecutive stages. This grouping pattern was consistent with the PLS-DA model based on the hypothesized stages. By integrating the abundance evolution trajectories of the three categories of components, the volatile compound evolution during processing could be tentatively divided into three proposed transformation windows:

Cycles 1–3 (early stage): Window of rapid dissipation of green-grassy odor. The color blocks corresponding to precursors gradually faded from dark blue-green, and the native low-boiling-point volatile compounds underwent rapid degradation and volatilization.

Cycles 4–6 (middle stage): Window of intermediate flavor compound generation. The color saturation of the blocks corresponding to intermediates increased significantly, and substances with roasted and malty aromas accumulated substantially, representing the core transformation stage where the flavor shifts from raw to processed.

Cycles 7–9 (late stage): Window of end-product accumulation and flavor stabilization. The color blocks of end-product components maintained a high-abundance state continuously from the sixth steaming and drying cycle onward and persisted through the ninth cycle, with the seventh cycle serving as the turning point for entering this stage. This indicated that the characteristic aroma quality of black ginseng had entered a stable phase, with the overall flavor profile largely finalized.

#### 3.3.3. Construction and Exploratory Evaluation of a Indicator for Aroma Progression

Drawing on the Rg3/Rb1 ratio approach [29], we proposed the M/C ratio (maltol/α-cedrol, tentatively identified and semi-quantified) to track aroma progression during processing. However, this indicator has only been evaluated on the present dataset and has not yet been independently validated across different batches, production regions, or processing conditions. The chemical significance of this indicator lies in the following: the numerator, maltol D, is a stable end-product of the Maillard reaction that continuously accumulates over the nine steaming and drying cycles; the denominator, α-cedrol, is a native sesquiterpene alcohol that is continuously depleted during repeated steaming. The M/C ratio integrates the two independent processes of end-product accumulation and precursor depletion into a single indicator, thereby comprehensively reflecting the overall transformation degree of the aroma system throughout processing.

The quantitative results showed that the M/C ratio remained within a low range of 0.002–0.007 during cycles 1–6, and increased sharply to 0.016–0.034 during cycles 7–9 (an increase of approximately one order of magnitude). This jump pattern highly corresponded to the three flavor transformation windows delineated previously, further confirming that the seventh steaming and drying cycle may represent a candidate transition point at which the aroma quality of black ginseng transitions from dynamic transformation to stabilization and finalization, and that the M/C ratio may serve as a candidate monitoring indicator for aroma progression.

### 3.4. Identification of Key Aroma-Contributing Components and Evolution of Aroma Categories

The previous section tentatively identified key differential markers from the perspective of chemical differences and clarified their roles in the Maillard reaction chain. However, the degree of difference in component content is not equivalent to its degree of sensory contribution to the actual aroma. To further identify the key components that theoretically estimated to contribute to the overall aroma of black ginseng at the sensory level, this study employed the relative odor activity value (ROAV) method [30] and systematically elucidated the evolution patterns of aroma categories during processing.

ROAV is an indicator used to estimate the degree of aroma contribution of a compound. Typically, ROAV ≥ 1 indicates that the compound directly contributes to the aroma, with higher ROAVs signifying greater contributions. In this study, the aroma thresholds of each compound were retrieved from the Leibniz-LSB@TUM Odorant Database. Among all 73 tentatively identified volatile components, threshold data for 28 compounds (38.36%) were available and used for ROAV calculation. Considering that traditional global normalization is susceptible to interference from differences in absolute component contents among different steaming and drying cycles, which may mask the relative ranking of aroma contributions within each processing stage, this study adopted an independent normalization approach for each individual steaming and drying cycle. Specifically, the compound with the highest odor activity value (C/T value) in a single steaming and drying sample was used as the reference and assigned an ROAV of 100, and the relative ROAV values of the remaining compounds in the same sample were calculated accordingly, thereby accurately reflecting the aroma contribution composition within each steaming and drying stage.

**Figure 9:**
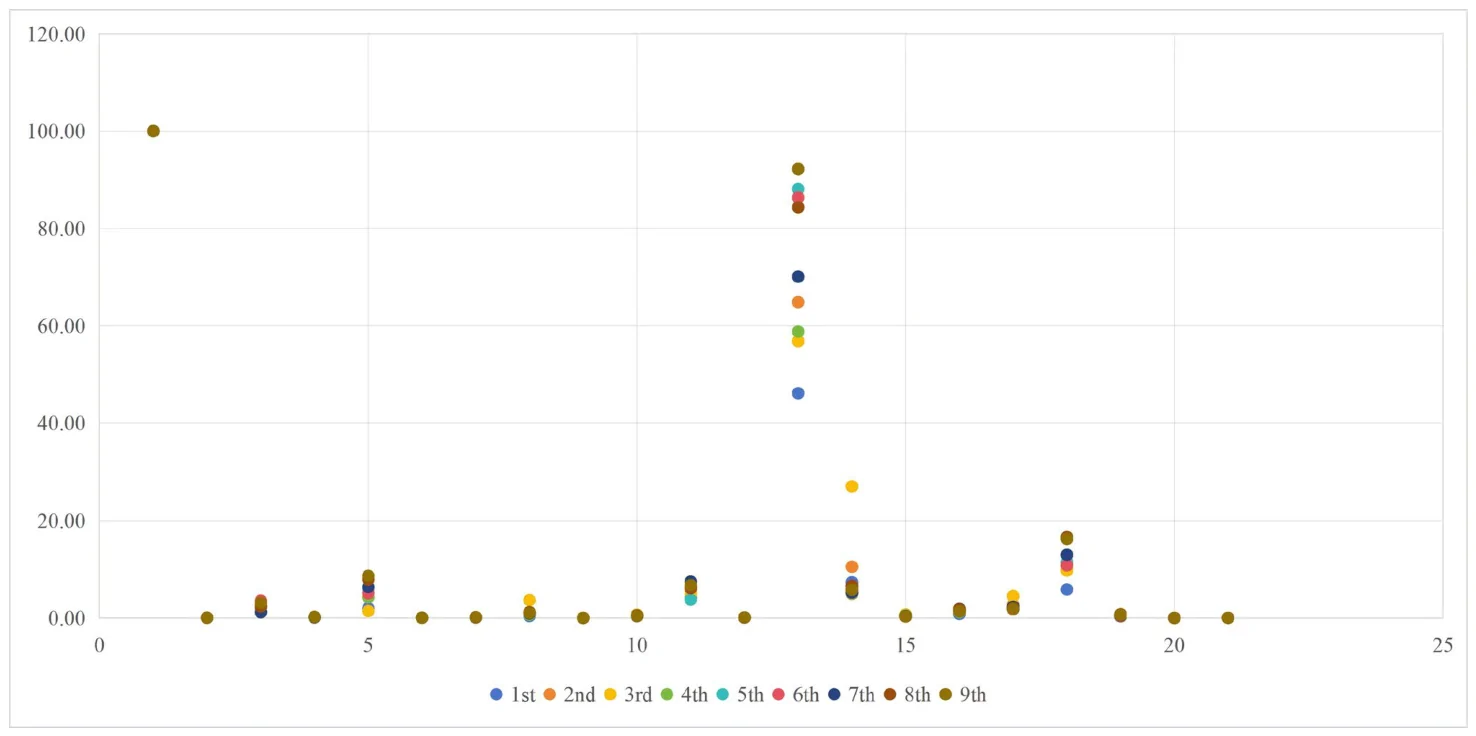
Scatter plot of ROAVs (relative odor activity values) of key aroma compounds in black ginseng across nine steaming cycles.

The ROAV analysis results (Figure 9, Table S3) showed that 2-methylbutanoic acid methyl ester (threshold 4.8 × 10^−5^ mg/kg, presenting an apple-like aroma) consistently exhibited an ROAV of 100 across all steaming and drying stages, indicating that it ranked as the most potent potential odorant relative to other compounds within each sample. Compounds such as 3-methylbutanal, butanal, 3-methylbutyl acetate, β-Myrcene, 2,3-Pentandione, 1-Hexanal, and n-Octanal D also exhibited ROAVs ≥ 1. Although the content of maltol increased more than 5-fold, its ROAV remained below 0.1 (threshold 5 mg/kg), and 2,3,5-trimethylpyrazine had an ROAV below 0.26 [31]; thus, both made limited direct contributions to the overall aroma. This indicates that VIP screening and ROAV analysis differ in their information dimensions: VIP values primarily characterize a component’s ability to discriminate chemical differences between groups, whereas ROAV provides a theoretical estimate of the degree of a compound’s contribution to the sensory aroma. From this perspective, based on theoretical ROAV estimates and subject to the semi-quantification limitations described above, the caramelized sweet and roasted aroma of black ginseng may result from the synergistic effect of multiple low-threshold components.

**Figure 10:**
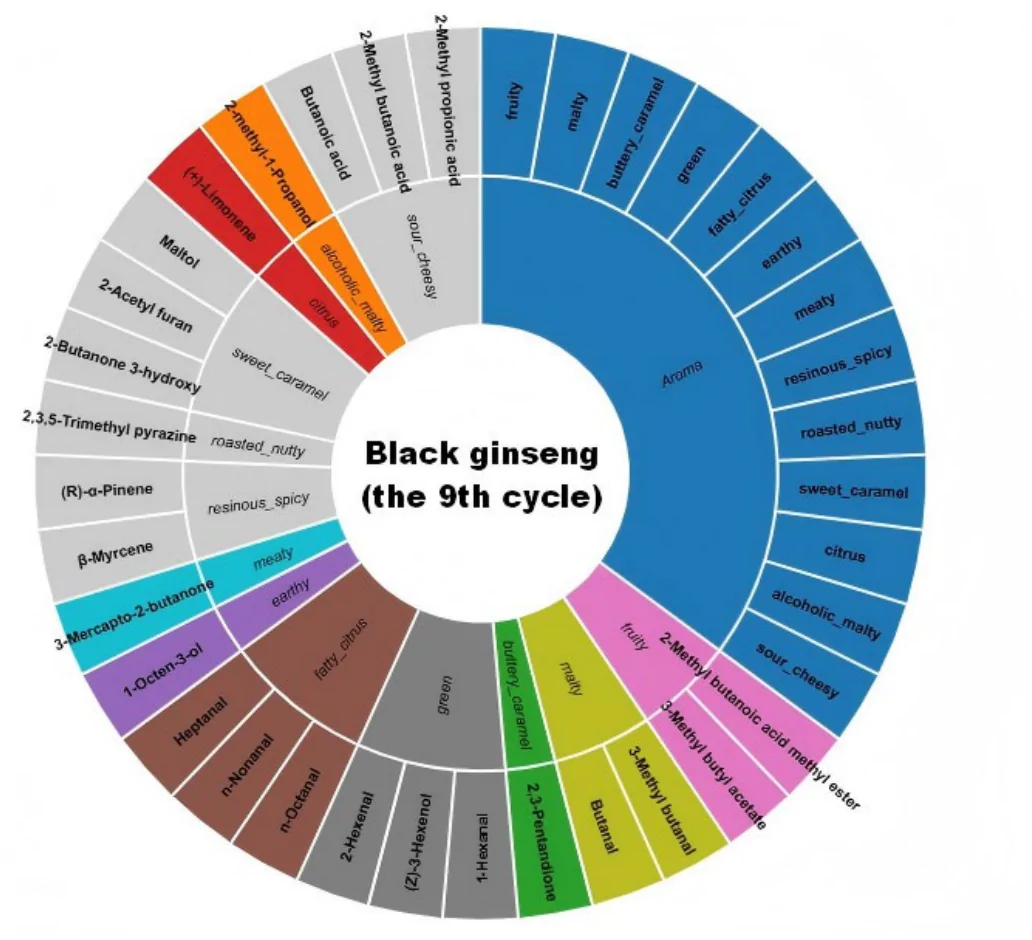
Evolution of aroma categories in black ginseng across nine steaming cycles. Note: Aroma wheel of the ninth steaming cycle, with sector sizes proportional to the ROAV contribution percentages of 13 aroma categories. The major contributing compounds of each category are listed within the corresponding sectors.

**Figure 11:**
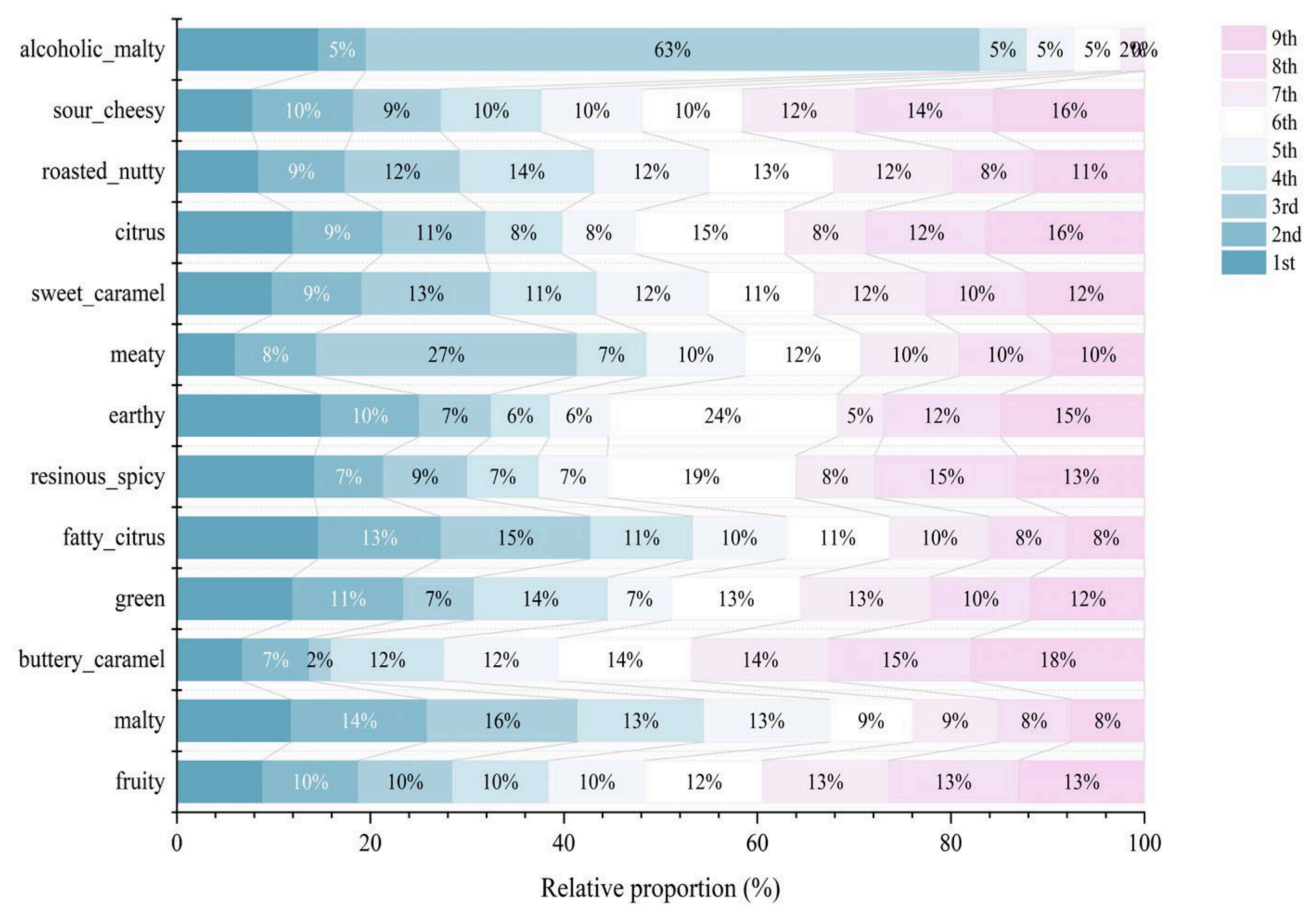
Distribution of contribution ratios of various aroma categories in black ginseng across different steaming–drying cycles based on ROAVs. Note: Relative contribution (%) of aroma categories within each steaming cycle based on ROAVs. For each cycle, the percentages of the 13 aroma categories sum to 100%.

The aroma wheel constructed based on ROAV (Figure 10) and the stacked bar chart of the relative contributions of each aroma category across the nine steaming and drying cycles (Figure 11) revealed that the relative contribution of fruity notes increased from 27.6% (first cycle) to 40.7% (ninth cycle), gradually becoming the dominant aroma category; malty notes peaked at 40.0% in the third cycle and then decreased to 19.6%; buttery-caramel notes increased from 1.7% to 13.5%; and green and fatty-citrus notes exhibited an overall decline. With increasing steaming and drying cycles, the aroma profile exhibited a pattern of shifting dominance: green/terpenic notes attenuated, fruity notes continuously increased, malty notes first increased and then decreased, and buttery-caramel notes steadily rose, ultimately forming a composite odor dominated by fruity notes, complemented by malty and buttery notes. This demonstrates that the aroma formation of black ginseng is the result of the combined action of multiple non-enzymatic reaction pathways, which interact with each other at different steaming and drying stages, and that the overall aroma formation process is not a uniform linear change but exhibits pronounced stage-specific fluctuations.

### 3.5. Evolution of Colorimetric Values During Processing and Their Correlation with Volatile Aroma Components

**Figure 12:**
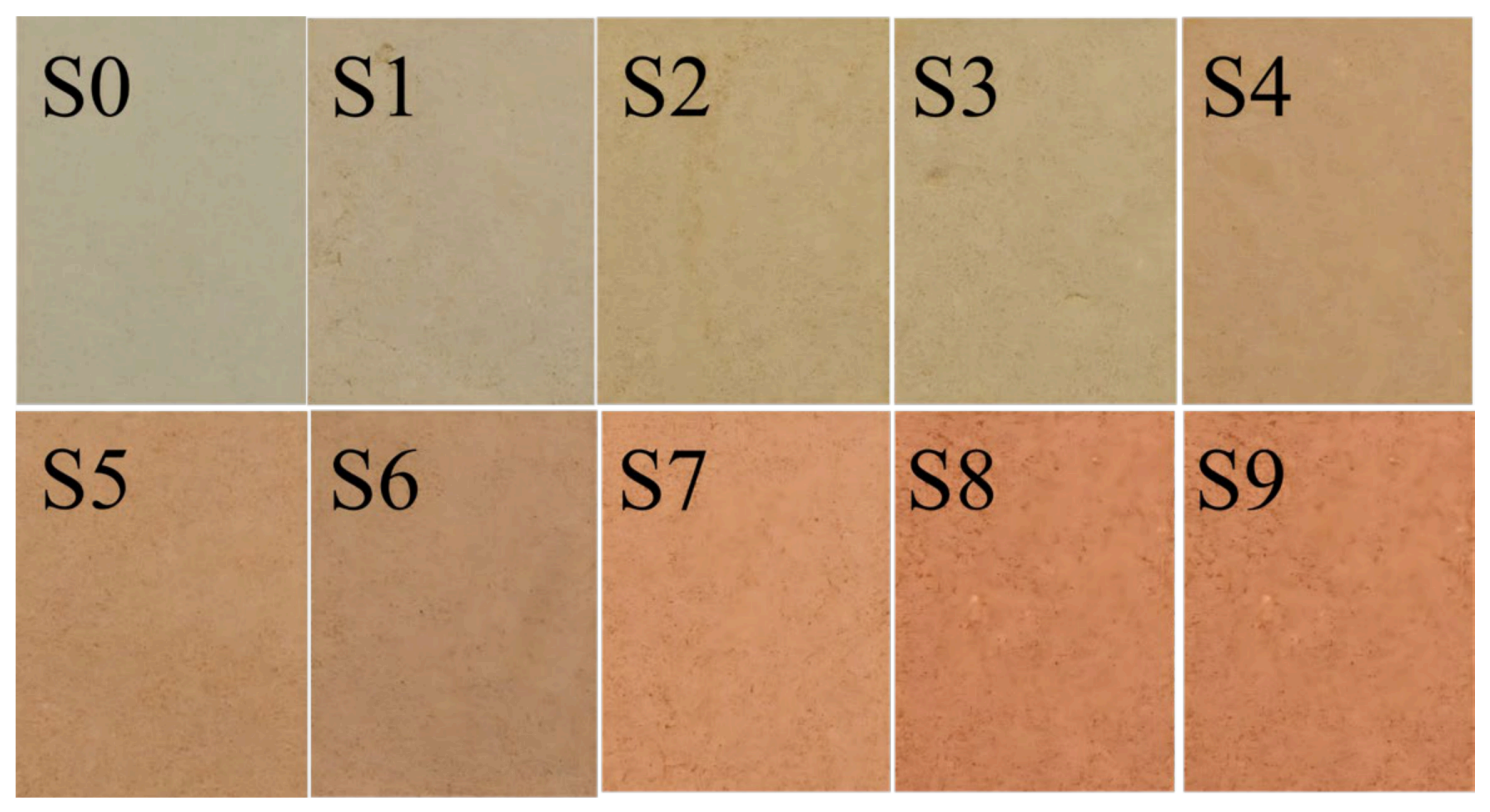
Powder samples of black ginseng across 0–9 steaming cycles.

During the nine-steaming and nine-drying process, color change is the most intuitive macroscopic manifestation, and its evolution pattern is highly synchronized with the internal material transformation processes. This study systematically determined the color parameters of samples at different steaming and drying cycles (Table 2) and simultaneously recorded the changes in appearance and morphology (Figure 12). The results showed that all color parameters exhibited significant and regular changes with increasing steaming and drying cycles (Figure 12): the lightness value L* decreased continuously from 54.82 (cycle 0) to approximately 10.5 by the 7th–8th cycles (cycles 1–9, *n* = 9); the redness value a* increased continuously from 2.15 to 94.97; the yellowness value b* turned negative after the 4th cycle, and its absolute value continuously increased until the 8th cycle; the CIELAB color vector magnitude increased from 56.67 to 265.49; and the sample appearance displayed a gradual transition from grayish-white to yellowish-brown and then to dark brown.

The critical turning points of the color changes were highly consistent with the aroma evolution process described above. During the 1st–4th cycles, L* decreased rapidly and a* increased rapidly, which completely overlapped with the accelerated depletion period of terpenes; during the 7th–9th cycles, all color parameters tended to plateau, consistent with the aroma maturation and stabilization stage. These findings are in agreement with the conclusions of studies on color changes in red and black ginseng during steaming by Qu Wenjia et al. [32] and Jiang Changpeng et al. [18].

The relationships between the color parameters L*, a*, and b*; CIELAB color vector magnitude (Eab); and the contents of nine chemical classes were examined using partial correlation analysis (Table S4). After controlling for steaming cycle number, only esters (Esters) remained significantly correlated with Eab (partial r = −0.783, *p* = 0.022, Bootstrap 95% CI: −0.975 to −0.009). No other chemical class showed a statistically significant partial correlation with Eab. After removing this time-driven trend, the net association between E*ab and Esters may reflect a nuanced regulatory relationship between color change and ester accumulation at a given degree of processing; however, the underlying mechanism requires further investigation. Under the conditions of this study, only E*ab retained a statistically significant association with ester content after controlling for steaming cycle number, suggesting that Eab may have potential as an auxiliary monitoring indicator for ester accumulation during black ginseng processing.

## 4. Discussion

The aroma evolution of black ginseng during the nine-steaming and nine-drying process exhibited distinct differences in the contribution weights of various pathways at different steaming stages. In the early stage (cycles 1–3), terpene degradation played a dominant role, with α-cedrol loss to 92.1% and green leaf volatiles such as (Z)-3-hexenol rapidly decreasing; the green-grassy odor dissipated, thereby freeing up olfactory space. In the middle stage (cycles 4–6), the Maillard reaction and lipid oxidation coexisted. The continuous supply of reducing sugars and amino acids [33] drove the persistent accumulation of furans such as maltol (increased 5.3-fold) and 2-acetylpyridine (increased 4.5-fold), while the decline in pyrazines after reaching their peak suggested possible secondary polymerization or thermal degradation. In the late stage (cycles 7–9), the accumulation of Maillard end-products and esterification reactions became predominant. Low-threshold esters such as methyl 2-methylbutyrate and isoamyl acetate emerged as potential candidate aroma contributors based on ROAV, and the lipid pathway completed a functional shift from oxidation to esterification. The report by Chen Libiao et al. [34] that reducing sugars first increased and then decreased, while amino nitrogen was continuously consumed, is consistent with the dynamic trends of Maillard products observed in this study. Cui et al. [35] also confirmed that terpenes dominate ginseng aroma and undergo changes during processing. The decoupling between chemical accumulation and sensory contribution suggests that the characteristic caramelized sweet and roasted aroma of black ginseng originates from the synergistic effects of multiple low-threshold compounds.

A core finding of this study is the evident decoupling between chemical accumulation and sensory contribution. High-abundance Maillard end-products such as maltol (ROAV < 0.001) and 2,3,5-trimethylpyrazine (ROAV < 0.27), although serving as sensitive chemical markers for distinguishing processing stages, contributed negligibly to the overall aroma quality compared to their concentrations. The compounds with the highest ROAVs, which are likely important for the fruity and sweet notes of black ginseng, are trace esters with extremely low odor thresholds. For example, 2-methylbutanoic acid methyl ester (threshold 4.8 × 10^−5^ mg/kg, apple-like aroma) consistently yielded the highest concentration-to-threshold (C/T) ratio in every steaming cycle and was therefore assigned an ROAV of 100 by definition. Since ROAV comparisons across cycles reflect relative ranking within each sample rather than absolute changes in perceived intensity, this result indicates that this ester ranked as the most potent potential odorant relative to other compounds within its respective sample. The characteristic caramelized sweet and roasted aroma of black ginseng thus appears to arise not from a single dominant component but from the synergistic interplay of multiple low-threshold, low-abundance odorants. Future flavor research should therefore pay closer attention to these low-threshold sensory-active molecules.

The stage-specific evolution of the aroma profile reached a plateau after the seventh steaming–drying cycle, indicating that repeated processing yields diminishing returns. This pattern parallels observations in black sesame, where quality indicators stabilized after multiple cycles [14,36], and in Polygonatum, where the pungent odor disappeared after six processing cycles [37]. Collectively, these findings suggest a general phenomenon of diminishing marginal utility in the traditional “nine-processing” technique. Based on the present dataset, the seventh steaming–drying cycle is proposed as a candidate transition point, beyond which the aroma composition enters a plateau phase. Whether this point corresponds to the optimal processing endpoint requires further evaluation that integrates sensory quality, bioactive compound profiles, product safety, and overall stability. Premature adoption of a shortened cycle should be avoided until independent validation is performed. Based on the theoretical ROAV profiles, the 5th–6th cycles are suggested to be associated with a more prominent roasted aroma, whereas cycles beyond the 7th are suggested to be associated with more pronounced fruity and caramelized sweet notes. The strong concurrent correlations between color parameters (a* and E*ab) and ester accumulation suggest that these color indices may serve as convenient empirical indicators for tracking aroma development, while the M/C ratio can function as a quantitative chemical candidate indicator for monitoring. These markers may collectively facilitate the transition of black ginseng processing from empirical judgment toward data-driven guidance.

Several methodological limitations must be considered. The ROAV analysis relies on aqueous aroma thresholds, and threshold data for some compounds were sourced from different studies and databases; variations in measurement conditions (e.g., medium, temperature) may introduce uncertainty. Thresholds were available for only 28 compounds, covering 38.36% of the tentatively identified volatiles, potentially missing certain unknown low-concentration yet highly potent odorants. Furthermore, the ROAV model assumes additive odor effects and cannot capture interactions such as synergism, masking, or suppression. The ROAV results should therefore be regarded as chemically approximate inferences of odor contribution rather than definitive sensory conclusions. In addition, the semi-quantification approach used a single internal standard (2-methyl-3-heptanone) without compound-specific response factors; consequently, the reported absolute concentrations (mg/kg) are internal-standard-equivalent estimates. Although fold changes for the same compound across processing cycles may remain informative if instrumental response is stable, ROAV rankings compare different compounds and can be affected by compound-specific response differences. Therefore, ROAV rankings should be interpreted as exploratory estimates. The absolute numerical values should also be interpreted with caution. At the statistical level, the markers should therefore be viewed as candidate compounds, and the observed correlations as concurrent patterns rather than causal relationships. Moreover, apart from the ROAV analysis, aroma perception was entirely based on chemical inference and lacked direct validation through quantitative descriptive analysis (QDA) with a trained sensory panel. Although three independent processing batches were employed, all roots originated from a single harvest batch and geographical region, which may limit the generalizability of the conclusions. In addition, because only three independent processing batches were available, batch was not incorporated as a random effect in the univariate models; batch-to-batch variability was assessed visually by PCA, and future studies with larger sample sizes should formally model batch effects.

## 5. Conclusions

This study employed HS-GC-IMS and multivariate chemometric methods to systematically characterize the dynamic evolution of volatile aroma compounds during the nine-steaming and nine-drying process of black ginseng. The results demonstrated that aroma formation is not a simple linear accumulation but rather a synergistic result driven by three pathways: the Maillard reaction, terpene transformation, and lipid oxidation–esterification. A total of 29 candidate differential compounds were screened, and their variation patterns revealed three distinct dynamic trends: decreasing, transient, and accumulating. ROAV analysis identified as the compounds with the highest relative odor activity values, suggesting they are potentially important contributors to the fruity and sweet aroma of black ginseng. In contrast, high-abundance Maillard products primarily served as chemical markers for processing stages, revealing a decoupling between chemical abundance and sensory contribution. After controlling for processing cycle, ester accumulation was significantly negatively correlated with the CIELAB color vector magnitude. No significant partial correlation was observed between ester accumulation and a* or b* individually. The constructed M/C ratio increased by approximately one order of magnitude after the seventh steaming and drying cycle, suggesting its potential as a candidate indicator for monitoring aroma progression. The seventh cycle represents a candidate transition point in this dataset, but independent validation is needed before it can be recommended as a definitive process endpoint. This study provides a chemical basis and analytical framework for the aroma quality control of black ginseng and the scientific elucidation of traditional processing techniques.

Future studies should incorporate authentic standard confirmation of key marker compounds, independent multi-batch validation of the M/C ratio across different production regions and harvest years, and direct sensory evaluation (GC–olfactometry, trained sensory panel) to bridge the gap between chemical profiles and perceived aroma quality.

## Figures and Tables

**Table 1:** Pattern-based grouping and plausible formation pathways of the 29 candidate differential.

Volatile Compounds	VIP	*q* (Adjusted *p*)	Pattern-Based Group/Plausible Formation Pathway
(Z)-3-Hexenol	1.77	8.2202 × 10^−13^	Group 1 (Decreasing)
(E)-2-Pentenal	1.751	2.5873 × 10^−7^	Group 1 (Decreasing)
4-Methyl-3-penten-2-one	1.548	5.7855 × 10^−10^	Group 1 (Decreasing)
2-Butanone	1.504	3.8482 × 10^−13^	Group 1 (Decreasing)
Butanoic acid	1.487	5.5218 × 10^−14^	Group 3 (Accumulating)
3-Furaldehyde D	1.402	1.6549 × 10^−16^	Group 2 (transient)
2,3,5-Trimethyl pyrazine	1.361	7.4465 × 10^−12^	Group 2 (transient)
Maltol D	1.282	4.5504 × 10^−17^	Group 3 (Accumulating)
2-Phenyl-1,3-dioxolane-4-methanol	1.278	3.0822 × 10^−6^	Group 2 (transient)
Ethyl E-2-hexenoate	1.248	7.7228 × 10^−9^	Group 2 (transient)
Isopentyl propanoate	1.23	9.7872 × 10^−14^	Group 3 (Accumulating)
2-Heptanol	1.221	5.3632 × 10^−12^	Group 2 (transient)
3-Methyl butanal D	1.217	3.5875 × 10^−14^	Group 2 (transient)
2-Acetyl pyridine	1.164	6.6728 × 10^−15^	Group 3 (Accumulating)
2-Methylbutanoic acid	1.153	7.4790 × 10^−12^	Group 1 (Decreasing)
1-Octene	1.147	3.9695 × 10^−7^	Group 2 (transient)
Butyl lactate	1.145	8.3344 × 10^−7^	Group 2 (transient)
Ethyl acetoacetate	1.135	4.7687 × 10^−7^	Group 3 (Accumulating)
Butanal	1.119	3.5515 × 10^−18^	Group 2 (transient)
2-Furancarboxaldehyde D	1.074	4.8791 × 10^−13^	Group 3 (Accumulating)
4,5-Dihydro-3(2H)-thiophenone	1.064	2.8875 × 10^−7^	Group 3 (Accumulating)
propyl acetate	1.063	3.2976 × 10^−8^	Group 3 (Accumulating)
2-Ethyl butanoic acid	1.06	1.5156 × 10^−12^	Group 3 (Accumulating)
1-(2-Furanyl)-ethanone D	1.058	3.6098 × 10^−13^	Group 3 (Accumulating)
5-Methyl furfural	1.052	2.9787 × 10^−13^	Group 3 (Accumulating)
α-Cedrol	1.03	5.6825 × 10^−15^	Group 1 (Decreasing)
4,5-Dimethylthiazole	1.018	2.1648 × 10^−10^	Group 3 (Accumulating)
3-Methyl-2-butenal D	1.015	6.5108 × 10^−10^	Group 3 (Accumulating)
2,3-Pentandione	1.003	4.8165 × 10^−12^	Group 3 (Accumulating)

**Table 2:** Changes in sample chromaticity values during ginseng steaming (Mean ± SD).

Steaming Times	L*	a*	b*	E*ab	Appearance Color
0	54.82 ± 0.6 a	2.15 ± 0.09 j	14.22 ± 0.09 c	56.67 ± 0.6 g	creamy white
1	50.08 ± 0.19 b	3.87 ± 0.08 i	18.6 ± 0.29 b	53.57 ± 0.28 h	grayish-yellow
2	27.77 ± 0.23 c	21.31 ± 0.41 h	32.55 ± 0.46 a	47.81 ± 0.13 i	yellowish-white
3	20.74 ± 0.26 d	35.3 ± 0.03 g	4.56 ± 0.08 d	41.19 ± 0.12 j	yellow-brown
4	12.82 ± 1.04 e	62.03 ± 0.44 f	−121.89 ± 0.35 e	137.37 ± 0.21 f	tan
5	12.02 ± 0.16 ef	69.68 ± 0.45 d	−182.77 ± 0.86 f	195.97 ± 0.65 e	fawn
6	12.11 ± 0.62 ef	86.43 ± 0.51 c	−190.84 ± 2.95 g	209.85 ± 2.65 c	yellowish-brown
7	10.45 ± 0.14 g	89.39 ± 0.42 b	−195.99 ± 0.42 h	215.67 ± 0.53 b	light brown
8	10.93 ± 0.04 fg	94.97 ± 0.58 a	−197.57 ± 0.56 h	219.48 ± 0.32 a	brown
9	12.16 ± 0.09 ef	88.48 ± 0.57 e	−180.27 ± 0.33 f	201.18 ± 0.061 d	brown

Note: The lowercase letters after the values indicate significant differences based on ANOVA followed by multiple comparisons.

## Data Availability

The processed data, compound identification tableand chemometric outputs generated in this study are openly available in the Figshare repository at [https://doi.org/10.6084/m9.figshare.33346380]. The raw HS-GC-IMS data files are available from the corresponding author upon reasonable request due to their large size and proprietary format.

## Supplementary Materials

The following supporting information can be downloaded at https://www.mdpi.com/article/10.3390/foods15173031/s1, Tables S1: Volatile compounds identified in black ginseng by HC-GC IMS, Table S2: Variation in the content of volatile components in black ginseng samples, Table S3: ROAV values of 28 compounds during the 1st to 9th drying cycles, Table S4: Partial correlation coefficients between color parameters and chemical classes, controlling for steaming cycle number (n = 9).

## Abbreviations

The following abbreviations are used in this manuscript:

BG	black ginseng
HS-GC-IMS	headspace–gas chromatography–ion mobility spectrometry
PLS-DA	partial least squares discriminant analysis
ANOVA	one-way analysis of variance
VIP	variable importance in projection
VOCs	volatile organic compounds
ROAV	relative odor activity value
