# Supplementary material for: Dynamic Evolution of Volatile Aroma Compounds in Black Ginseng During Nine-Steaming and Nine-Drying Based on HS-GC-IMS and Chemometrics
Source: Foods. 2026 Aug 27;15(17):3031. doi: 10.3390/foods15173031 (PMC13565742; doi:10.3390/foods15173031)
Supplement: Supplementary file 1 [file foods-15-03031-s001.zip › foods-4453794-supplementary.pdf]

Table S1. Volatile compounds identified in black ginseng by HC-GC-IMS

Category	Compound	CAS#	Formula	MW	RI	Rt [sec]	Dt [a.u.]	Odor description
Terpenoids	(R)-α-pinene	C7785708	C10H16	136.2	935.1	484.875	1.2183	Terpenic, Mint, Pine
Terpenoids	(+)-Limonene	C138863	C10H16	136.2	1026.7	567.413	1.2206	lemon, sweet, orange, pine oil
Terpenoids	(E)- β-Farnesene	C18794848	C15H24	204.4	1458.1	1336.635	1.44091	wood, citrus, sweet, herb
Terpenoids	(E),(E)-α-farnesene	C502614	C15H24	204.4	1496.3	1442.051	1.43845	citrus herbal lavender bergamot myrrh neroli green
Terpenoids	Camphene	C79925	C10H16	136.2	951.3	497.481	1.21231	woody, camphor
Terpenoids	Carvone	C99490	C10H14O	150.2	1265.6	911.94	1.29587	spearmint
Terpenoids	α-Terpinolene	C586629	C10H16	136.2	1090	643.406	1.22144	fresh woody sweet pine citrus
Terpenoids	β-mircene D	C123353	C10H16	136.2	997.9	535.852	1.71251	must, spice, balsamic
Terpenoids	β-myrcene M	C123353	C10H16	136.2	996.8	534.625	1.22178	must, spice, balsamic
Terpenoids	β-Pinene D	C127913	C10H16	136.2	980.2	520.698	1.29247	resin, green
Terpenoids	β-Pinene M	C127913	C10H16	136.2	980.6	521.037	1.21674	resin, green
Terpenoids	β-Pinene P	C127913	C10H16	136.2	980.8	521.264	1.64008	resin, green
Terpenoids	γ-Terpinene	C99854	C10H16	136.2	1048.4	592.319	1.21234	oil, wood, terpenes, lemon, lime, herbs
Terpenoids	α-Cedrol	C77532	C15H26O	222.4	1622.8	1853.864	1.44461	cedar wood, sweet
Ketones	1-(2-Furanyl)-ethanone D	C1192627	C6H6O2	110.1	914.6	469.4	1.44091	fatty, sweet, caramel, nutty, tobacco
Ketones	1-(2-Furanyl)-ethanone M	C1192627	C6H6O2	110.1	914.2	469.087	1.11622	fatty, sweet, caramel, nutty, tobacco
Ketones	2,3-pentandione	C600146	C5H8O2	100.1	692.6	250.077	1.22834	sweet, cream, caramel, nuts, cheese
Ketones	2-Butanone	C78933	C4H8O	72.1	594.5	175.319	1.2514	fruity , camphor
Ketones	2-butanone 3-hydroxy	C513860	C4H8O2	88.1	730.3	292.941	1.33463	butter, cream
Ketones	3-Heptanone	C106354	C7H14O	114.2	889.5	450.688	1.2283	Fruity, Grass, Oil
Ketones	3-Mercapto-2-butanone	C40789988	C4H8OS	104.2	816.1	390.925	1.45268	onion, sulfur
Ketones	4,5-dihydro-3(2H)-thiophenone	C1003049	C4H6OS	102.2	961.1	505.236	1.44517	onion, garlic, meaty, vegetable, cream
Ketones	4-Methyl-3-penten-2-one	C141797	C6H10O	98.1	798.6	377.908	1.44497	spice, earth, green
Ketones	Maltol D	C118718	C6H6O3	126.1	1128	693.813	1.52326	sweet, fruity, caramel, toasted bread
Ketones	Maltol M	C118718	C6H6O3	126.1	1133.6	701.646	1.14325	sweet, fruity, caramel, toasted bread
Aldehydes	(E)-2-Heptenal D	C18829555	C7H12O	112.2	961.8	505.79	1.66827	spicy, green vegetables, fresh, fatty
Aldehydes	(E)-2-Heptenal M	C18829555	C7H12O	112.2	961.8	505.79	1.25693	picy, green vegetables, fresh, fatty
Aldehydes	(E)-2-Octenal	C2548870	C8H14O	126.2	1058.1	603.899	1.33235	fresh cucumber, fatty, green herbal, banana, green leaf
Aldehydes	(E)-2-Pentenal	C1576870	C5H8O	84.1	767.5	342.544	1.35799	potato, peas

Aldehydes	1-hexanal	C66251	C6H12O	100.2	799.2	378.369	1.56497	fresh, green, fat, fruity
Aldehydes	2-Formyl-1-methylpyrrole	C1192581	C6H7NO	109.1	1007.4	545.999	1.50144	nut
Aldehydes	2-furancarboxaldehyde D	C98011	C5H4O2	96.1	840.5	409.881	1.32889	sweet, woody, almond, bready
Aldehydes	2-furancarboxaldehyde M	C98011	C5H4O2	96.1	842.9	411.759	1.08538	sweet, woody, almond, bready
Aldehydes	2-Hexenal	C505577	C6H10O	98.1	849.2	416.828	1.17481	sweet almonds, fruity, green leaves, apples, plums, vegetables
Aldehydes	3-Furaldehyde D	C498602	C5H4O2	96.1	821.2	394.859	1.36337	Sweet, nutty, and almond notes, accompanied by caramel, toasted bread, and woody undertones, with subtle floral and fruity hints and a slightly bitter finish.
Aldehydes	3-Furaldehyde M	C498602	C5H4O2	96.1	820.3	394.108	1.11124	Sweet, nutty, and almond notes, accompanied by caramel, toasted bread, and woody undertones, with subtle floral and fruity hints and a slightly bitter finish.
Aldehydes	3-Methyl butanal D	C590863	C5H10O	86.1	652.2	215.746	1.19825	chocolate, fat
Aldehydes	3-Methyl butanal M	C590863	C5H10O	86.1	663.5	224.684	1.40583	chocolate, fat
Aldehydes	3-Methyl-2-butenal D	C107868	C5H8O	84.1	785.2	368.196	1.36121	fruity
Aldehydes	3-Methyl-2-butenal M	C107868	C5H8O	84.1	787.5	369.886	1.09184	fruity
Aldehydes	5-methyl furfural	C620020	C6H6O2	110.1	970.4	512.773	1.12302	spices, caramel wood
Aldehydes	butanal	C123728	C4H8O	72.1	606.5	183.039	1.29352	pungent, fruity, green leaf
Aldehydes	Heptanal	C111717	C7H14O	114.2	903	460.896	1.70001	fresh, aldehyde, fatty, green herbs, wine, fruity
Aldehydes	n-Nonanal	C124196	C9H18O	142.2	1112.7	673.053	1.47122	rose, citrus, strong oily
Aldehydes	n-Octanal D	C124130	C8H16O	128.2	1006.4	544.978	1.8269	aldehyde, waxy, citrus, orange, fruity, fatty
Aldehydes	n-Octanal M	C124130	C8H16O	128.2	1008	546.68	1.40144	aldehyde, waxy, citrus, orange, fruity, fatty
Aldehydes	n-pentanal	C110623	C5H10O	86.1	706.1	264.704	1.42789	green grassy, faint banana, pungent
Esters	(Z)-3-Hexen-1-ol benzoate	C25152856	C13H16O2	204.3	1550.9	1606.975	1.44091	green
Esters	2-methyl butanoic acid methyl ester	C868575	C6H12O2	116.2	781.7	363.739	1.18845	apple
Esters	3-Methyl butyl acetate	C123922	C7H14O2	130.2	871.6	435.377	1.30844	sweet, banana, fruity
Esters	acetic acid ethyl ester	C141786	C4H8O2	88.1	615.9	189.337	1.34065	fresh, fruity, sweet, grassy
Esters	acetic acid pentyl ester	C628637	C7H14O2	130.2	908.8	465.149	1.30959	bananas, apples, pears
Esters	propyl acetate	C109604	C5H10O2	102.1	690.5	247.843	1.15915	fruity, pear
Esters	Butyl formate	C592847	C5H10O2	102.1	743.9	310.209	1.20628	plum
Esters	Butyl lactate	C138227	C7H14O3	146.2	1014.7	554.027	1.2689	cream, milk
Esters	Ethyl acetoacetate	C141979	C6H10O3	130.1	941.8	490.075	1.60009	fruity
Esters	ethyl E-2-hexenoate	C27829727	C8H14O2	142.2	1057.3	602.877	1.81599	fruity, vegetable

Esters	Isopentyl propanoate	C105680	C8H16O2	144.2	968.3	511.053	1.35453	fruity
Alcohols	(Z)-3-hexenol	C928961	C6H12O	100.2	856.3	422.647	1.51604	green, herb
Alcohols	1-Octen-3-ol	C3391864	C8H16O	128.2	978.7	519.508	1.73039	mushroom, lavender, rose, hay
Alcohols	1-Propanol, 2-methyl-	C78831	C4H10O	74.1	633.8	201.932	1.37173	fresh, alcoholic, leather
Alcohols	2-(2-methoxyethoxy)ethanol	C111773	C5H12O3	120.1	987.2	526.57	1.15783	Aromatic odor
Alcohols	2,3-Butanediol	C513859	C4H10O2	90.1	804	381.903	1.36337	sweet, fruity, butter
Alcohols	2-Butoxyethanol	C111762	C6H14O2	118.2	903.5	461.281	1.20213	pungent, ether
Alcohols	2-Heptanol	C543497	C7H16O	116.2	896.7	456.278	1.36798	mushroom、melon
Alcohols	2-phenyl-1.3-dioxolane-4-methanol	C1708390	C10H12O3	180.2	967.1	510.052	1.15042	sweet berries , bitter almonds
Acids	(E)-2-Butenoic acid	C107937	C4H6O2	86.1	895	455.088	1.11049	pungent
Acids	2-Ethylbutanoic acid	C88095	C6H12O2	116.2	924.5	476.795	1.24794	acidic, Fruit
Acids	2-Heptenoic acid	C18999285	C7H12O2	128.2	1186.6	779.517	1.40964	special
Acids	2-Methyl butanoic acid	C116530	C5H10O2	102.1	889.2	450.445	1.47331	pungent and spicy cheese, fruity）
Acids	2-Methyl propionic acid	C79312	C4H8O2	88.1	788.8	370.828	1.15331	yogurt, rancid cream
Acids	3-Methyl valeric acid	C105431	C6H12O2	116.2	946.4	493.603	1.27366	sour, herbal, slight green
Acids	Butanoic acid	C107926	C4H8O2	88.1	779.5	360.356	1.3899	strong acetic acid, cheese, butter, fruity
Pyrazines	2, 3, 5-trimethyl pyrazine	C14667551	C7H10N2	122.2	1000.1	538.162	1.16573	roasted potato, peanut, cocoa, chocolate
Pyrazines	2-Ethyl-6-methyl pyrazine	C13925036	C7H10N2	122.2	992.3	530.779	1.17803	nutty, roast, sweet
Pyrazines	2-ethyl pyrazine	C13925003	C6H8N2	108.1	938.4	487.412	1.12627	nutty, mouldy, woody, potato, earthy, roast, meat, fishy, cocoa
Furan	2-Acetyl furan	C1192627	C6H6O2	110.1	903.3	461.139	1.44355	fatty, sweet, caramel, nutty, tobacco
Furan	2-Pentyl furan	C3777693	C9H14O	138.2	996.8	534.625	1.25372	bean, fruity, earthy, green, vegetable
Phenols	p-tert-Butylphenol	C98544	C10H14O	150.2	1309.6	995.213	1.32332	phenolic
Others	1-Octene	C111660	C8H16	112.2	782.6	365.1	1.45957	Gasoline
Others	2-acetylpyridine	C1122629	C7H7NO	121.1	1034.3	575.971	1.12143	popcorn, nutty, tobacco
Others	Methyl eugenol	C93152	C11H14O2	178.2	1415.9	1229.232	1.45479	sweet, spiciness, clove
Others	allyl isothiocyanate	C57067	C4H5NS	99.2	889.5	450.688	1.08747	sulfur, pungent, garlic
Others	4,5-dimethylthiazole	C3581917	C5H7NS	113.2	936.2	485.763	1.49314	Green, Nut, Roast

Note: "M" and "D" suffixes denote monomer and dimer ion signals, respectively. Both forms represent the same chemical compound and were merged for unique compound counting (74 unique compounds from 85 detected signals). For quantitative analysis, peak volumes of M and D forms were summed to obtain the total concentration. Retention indices (RI) were calculated from C4 – C9 n-ketone standards. Reference RI values were obtained from the NIST 2020 Retention Index Library.

Table S2 Variation in the content of volatile components in black ginseng samples

No.	compounds	1st	2nd	3rd	4th	5th	6th	7th	8th	9th
1	(R)-α-Pinene	3.33±0.045a	1.03±0.047f	2.06±0.061bc	1.37±0.055de	1.42±0.03d	2.14±0.064b	1.07±0.032ef	1.19±0.037def	1.76±0.148c
2	β-Pinene P	1±0.025c	0.35±0.015e	0.6±0.028de	0.45±0.009e	0.36±0.015e	2.35±0.129a	0.37±0.043e	0.77±0.047cd	1.36±0.085b
3	(E)-β-Farnesene	5.86±0.091a	2.71±0.113cd	4.61±0.237b	4.02±0.13b	2.5±0.143d	3.1±0.03c	2.23±0.076de	1.85±0.026e	3.19±0.181c
4	(E),(E)-α-Farnesene	25.47±0.18a	12.29±0.557ef	21.64±0.386b	17.61±0.179c	11.59±0.111f	14.22±0.088d	8.67±0.269g	6.88±0.121h	13.32±0.965de
5	α-Terpinolene	0.67±0.015de	0.4±0.007e	0.62±0.021de	0.56±0.013de	0.7±0.026d	3.54±0.114a	0.5±0.025de	1.51±0.038c	2.72±0.122b
6	β-Myrcene	2.65±0.033b	1.15±0.053d	1.5±0.029c	1.15±0.028d	1.01±0.018d	3.63±0.075a	0.97±0.023d	1.61±0.038c	2.45±0.151b
7	γ-Terpinene	1.46±0.013a	0.32±0.018d	0.31±0.007d	0.44±0.012b	0.42±0.017bc	0.39±0.003bcd	0.42±0.023bc	0.34±0.009cd	0.45±0.032b
8	Camphene	2.88±0.043a	0.71±0.038efg	1.4±0.075b	1.03±0.033cd	0.92±0.023cde	1.12±0.066c	0.64±0.046fg	0.49±0.027g	0.82±0.044def
50	α-Cedrol	81.3±1.325a	51.33±1.53de	73.61±1.79b	58.68±1.353c	46.87±1.866e	54.28±1.432cd	26.45±0.717f	28.63±0.566f	46.85±0.925e
9	Carvone	0.45±0.026b	0.41±0.027b	2.52±0.124a	0.43±0.029b	0.47±0.04b	0.61±0.012b	0.46±0.024b	0.38±0.003b	2.65±0.18a
10	(+)-Limonene	0.92±0.023b	0.57±0.009de	0.72±0.024c	0.57±0.007de	0.52±0.012e	1.18±0.025a	0.62±0.015d	0.85±0.012b	1.26±0.015a
11	1-(2-Furanyl)-ethanone D	0.96±0.007d	1.12±0.027d	0.85±0.02d	1.89±0.046c	2.22±0.073c	2.76±0.101b	2.73±0.078b	2.79±0.038b	3.89±0.251a
12	2,3-Pentandione	8.32±0.152e	11.49±0.242d	2.64±0.192f	13.47±0.197d	16.09±0.495bc	16.77±0.191b	17.22±0.311b	17.35±0.217b	22.1±1.832a
13	2-Butanone	14.04±0.081bc	10.58±0.267f	8.59±0.211g	14.95±0.167b	14.15±0.186bb	17.1±0.175a	12.92±0.46cd	11.37±0.296ef	12.36±0.197de
19	Maltol D	0.18±0.003f	0.22±0.017ef	0.21±0.006ef	0.26±0.006de	0.31±0.009cd	0.33±0.017c	0.43±0.033b	0.96±0.019a	0.95±0.015a

14	2-Butanone 3-hydroxy	28.07±0.515d	38.57±1.006a	33.69±1.31b	32.89±0.724bc	31.63±1.153bcd	29.21±0.293cd	28.63±0.575d	31.5±0.673bcd	31.58±0.854bcd
15	3-Heptanone	0.43±0.007c	0.39±0.006c	0.8±0.015a	0.45±0.01c	0.61±0.025b	0.58±0.006b	0.45±0.035c	0.44±0.007c	0.45±0.023c
16	3-Mercapto-2-butanone	1.22±0.015g	1.58±0.048f	5.25±0.09a	1.69±0.073ef	2.35±0.085b	2.51±0.129bc	1.89±0.026def	2.05±0.059cd	1.99±0.122de
17	4,5-Dihydro-3(2H)-thiophen one	2±0.02e	2.41±0.113cd	1.59±0.05e	2.55±0.072bcd	2.5±0.13cd	2.65±0.067bc	3.03±0.212ab	3.32±0.041a	3.27±0.25a
18	4-Methyl-3-penten-2-one	0.81±0.029cd	0.7±0.035de	0.96±0.017bc	0.79±0.038cde	1.57±0.058a	1.14±0.042b	0.71±0.102de	0.63±0.012de	0.57±0.035e
20	(E)-2-Heptenal D	3.53±0.082a	3.2±0.137ab	3.33±0.136ab	3.12±0.049abc	2.74±0.084ce	3.37±0.058ab	3.43±0.041ab	3.06±0.096bce	2.66±0.09e
21	(E)-2-Octenal	1.63±0.007c	1.54±0.062cd	1.37±0.04d	1.65±0.047c	1.55±0.046cd	2.07±0.058a	2.08±0.032a	1.91±0.019ab	1.71±0.049bc
22	(E)-2-Pentenal	2.57±0.021de	3.04±0.142bcd	3.12±0.15abc	3.56±0.047a	3.3±0.077abc	3.42±0.08ab	2.84±0.044cde	2.4±0.086e	2.42±0.137e
23	1-Hexanal	10.72±0.106b	9.39±0.416bc	5.55±0.141d	12.81±0.315a	6.3±0.293d	12.36±0.248a	12.38±0.391a	8.35±0.137c	10.53±0.21b
24	2-Formyl-1-methylpyrrole	1.96±0.013b	1.82±0.068b	3.46±0.213a	1.79±0.088b	1.81±0.058b	1.87±0.046b	1.74±0.035b	1.86±0.012b	1.96±0.078b
25	2-Furancarboxaldehyde D	10.09±0.064e	12±0.476e	11.21±0.251e	17.59±0.365c	15.43±0.498d	20.82±0.699ab	20.2±0.309b	18.2±0.095c	22.16±0.707a
26	2-Hexenal	5.73±0.113c	6.22±0.174bc	5.4±0.09c	6.07±0.185c	7.14±0.244ab	7.29±0.211a	7.07±0.058ab	7.75±0.015a	7.62±0.476a
27	3-Furaldehyde D	0.74±0.015g	1.47±0.047f	2.21±0.105cd	1.97±0.085de	6.63±0.246a	3.72±0.059b	1.8±0.032ef	2.4±0.045c	2.06±0.05cde
28	3-Methyl butanal D	19.77±0.389c	18±0.422c	10.85±0.327d	19.02±0.26c	24.67±0.575b	29.29±0.581a	19.39±0.207c	19.05±0.152c	24.13±0.672b
29	3-Methyl-2-butenal D	2.18±0.075f	2.8±0.078e	2.81±0.067e	3.3±0.041de	3.75±0.104cd	4.78±0.185a	4.42±0.179ab	4.1±0.044bc	3.65±0.236cd
30	5-Methyl furfural	0.31±0.012e	0.41±0.018e	0.86±0.019c	0.59±0.02d	0.68±0.031d	0.82±0.044c	0.87±0.006bc	0.99±0.012ab	1.08±0.036a
31	Butanal	6.81±0.096b	6.35±0.147b	11.22±0.191a	3.49±0.134cd	3.19±0.062d	3.81±0.162c	3.17±0.04d	3.23±0.037cd	3.3±0.123cd

32	Heptanal	3.92±0.11a	2.29±0.109bc	2.1±0.03c	2.41±0.08b	1.49±0.042d	1.5±0.035d	1.69±0.044d	1.14±0.023e	1.17±0.035e
33	n-Nonanal	2.48±0.007c	2.79±0.09bc	1.86±0.076d	2.72±0.077c	3.16±0.095ab	3.42±0.159a	3.58±0.052a	2.79±0.057bc	2.43±0.089c
34	n-Octanal D	9.02±0.104a	6.4±0.155c	7.29±0.164b	6.42±0.177c	4.88±0.105d	5.17±0.167d	5.39±0.07d	3.77±0.077e	4.21±0.115e
35	n-Pentanal	7.4±0.195a	6.29±0.178c	4.88±0.18de	5.57±0.149cd	5.96±0.087bc	7.34±0.16a	6.56±0.121b	6.5±0.077b	4.52±0.107e
36	2-Methyl butanoic acid methyl ester	5.14±0.1a	3.33±0.116c	2.29±0.066e	3.88±0.047b	3.36±0.038c	4.07±0.072b	3.32±0.106c	2.71±0.02d	3.14±0.075c
37	3-Methyl butyl acetate	45.13±0.87e	55.68±1.08d	33.44±1.761f	62.86±1.798bc	57.76±0.827cd	66.07±1.903b	64.57±0.636b	67.7±1.086b	76.45±0.265a
38	Acetic acid ethyl ester	4.81±0.06b	6.17±0.117b	33.64±1.347a	2.29±0.09c	2.12±0.079c	2.52±0.195c	1.95±0.023c	1.84±0.042c	1.87±0.052c
39	Acetic acid pentyl ester	1.79±0.021e	2.73±0.098d	1.28±0.015f	3.29±0.088c	2.78±0.076d	3.42±0.084bc	3.32±0.09c	3.69±0.047b	4.64±0.189a
40	propyl acetate	16.8±0.226de	19.33±0.547cd	15.17±0.326e	22.54±0.964bc	23.59±0.461ab	22.85±0.61ab	22.4±0.356bc	20.57±0.251bc	26.09±1.42a
41	Allyl isothiocyanate	0.47±0.013cd	0.38±0.01ef	0.23±0.01g	0.45±0.021de	0.38±0.019f	0.56±0.003ab	0.53±0.02abc	0.51±0.003bcd	0.59±0.019a
42	Butyl formate	11.55±0.278e	13.35±0.121d	18.69±0.59a	12.95±0.453de	12.88±0.187de	13.61±0.207cd	13.36±0.091d	15.18±0.04bc	16.67±0.448b
43	Butyl lactate	0.96±0.012bcd	0.86±0.006cd	0.82±0.02d	1.35±0.092a	0.91±0.023cd	0.86±0.006cd	1.08±0.045bc	1.31±0.094a	1.14±0.038ab
44	Ethyl acetoacetate	1.18±0.042de	1.15±0.013e	1.17±0.048e	1.2±0.025de	1.28±0.032cde	1.36±0.029bcd	1.41±0.044bc	1.47±0.026ab	1.59±0.052a
45	Ethyl E-2-hexenoate	0.55±0.02b	0.52±0.019b	0.68±0.025a	0.44±0.012c	0.49±0.009bc	0.55±0.021b	0.67±0.003a	0.66±0.013a	0.5±0.006bc
46	Isopentyl propanoate	0.56±0.007f	0.64±0.021ef	0.64±0.02ef	0.65±0.02ef	0.72±0.009de	0.81±0.012cd	0.87±0.034c	1.22±0.007b	1.43±0.06a
47	(Z)-3-Hexenol	1.26±0.009bc	1.29±0.045bc	1.44±0.073ab	1.1±0.032cd	0.98±0.015d	1.17±0.056cd	1.43±0.09ab	1.63±0.012a	1.54±0.049a
48	(Z)-3-Hexen-1-ol benzoate	63.11±0.539a	29.67±1.145e	54.56±1.436b	45.6±1.018c	29.18±0.498e	34.44±1.375d	20.49±0.3f	16.32±0.149g	33.01±1.317de

49	1-Propanol, 2-methyl-	2.22±0.019b	0.23±0.009c	8.23±0.235a	0.25±0.012c	0.24±0.018c	0.31±0.025c	0.18±0.006c	0.11±0.006c	0.15±0.012c
51	2-(2-Methoxyethoxy)ethanol	0.86±0.009bc	0.84±0.023bc	0.67±0.009d	0.75±0.024cd	1.05±0.047a	0.77±0.055cd	0.96±0.009ab	0.9±0.012b	0.84±0.019bc
52	2,3-Butanediol	0.65±0.003f	1.51±0.025e	2.41±0.172d	1.34±0.057e	5.52±0.12a	3.02±0.052c	2.16±0.067d	3.96±0.055b	3.16±0.078c
53	2-Butoxyethanol	3.17±0.039e	3.82±0.055d	3.74±0.186d	3.41±0.127de	4.35±0.172c	5.49±0.216a	5.01±0.082ab	5.2±0.019ab	4.77±0.061bc
54	2-Heptanol	1.32±0.012d	2.4±0.075c	1.45±0.056d	3.32±0.111a	2.87±0.075b	2.09±0.114c	2.83±0.101b	1.52±0.033d	2.08±0.092c
55	2-Phenyl-1.3-dioxolane-4-methanol	1.21±0.015c	0.9±0.015d	1.24±0.07bc	1.15±0.039c	1.39±0.087ab	1.44±0.022a	1.09±0.026c	1.06±0.028cd	1.2±0.037c
56	Methyl eugenol	8.75±0.026a	4.71±0.107e	7.22±0.131b	7.2±0.025b	4.26±0.095ef	5.62±0.16d	3.83±0.012fg	3.66±0.098g	6.3±0.222c
57	p-Tert-Butylphenol	3.3±0.071a	1.41±0.041cd	2.17±0.093b	1.56±0.061c	1.29±0.012d	1.29±0.018d	1.23±0.017d	1±0.01e	1.34±0.018d
58	1-Octen-3-ol	1.37±0.089b	0.49±0.015de	0.65±0.015d	0.51±0.009de	0.39±0.01e	2.18±0.073a	0.35±0.015e	0.86±0.009c	1.42±0.019b
59	(E)-2-Butenoic acid	0.66±0.009c	0.77±0.035b	0.51±0.033d	0.94±0.04a	0.53±0.007d	0.35±0.023e	0.75±0.015bc	0.26±0.013f	0.39±0.013e
60	2-Ethyl butanoic acid	0.86±0.015e	1.35±0.02d	1.06±0.003e	1.65±0.066c	2.09±0.075b	2.39±0.123a	2.23±0.04ab	2.47±0.023a	2.45±0.11a
61	2-Heptenoic acid	2.24±0.032b	2.02±0.06bc	1.76±0.082c	2.03±0.064bc	2.24±0.099b	2.63±0.138ab	2.77±0.047a	2.16±0.019b	2.22±0.05b
62	2-Methylbutanoic acid	0.39±0.003f	0.42±0.006f	0.48±0.032e	0.55±0.007d	0.61±0.01bc	0.66±0.01ab	0.71±0.015a	0.6±0cd	0.64±0bc
63	2-Methylpropionic acid	0.96±0.031e	1.19±0.017d	1.8±0.088b	1.52±0.059c	1.61±0.062bc	1.51±0.01c	1.56±0.009c	1.76±0.029b	2.15±0.076a
64	3-Methyl valeric acid	3.72±0.029a	2.9±0.116bcd	2.26±0.08e	3.18±0.115bc	2.73±0.1cd	3.28±0.115b	2.99±0.021bcd	2.78±0.052cd	3.01±0.065bcd
65	Butanoic acid	2.26±0.015d	2.68±0.088bc	2.16±0.079d	2.1±0.091d	2.22±0.051d	2.41±0.095cd	2.95±0.055b	4.22±0.044a	4.09±0.076a
66	2, 3, 5-Trimethyl pyrazine	0.76±0.009e	1.44±0.041bc	1.06±0.056d	2.11±0.067a	2.02±0.068a	1.17±0.072cd	1.62±0.104b	0.71±0.024e	1.1±0.032d
67	2-Ethyl-6-methyl pyrazine	1.75±0.013abc	1.77±0.049abc	1.73±0.113abc	1.78±0.042ab	1.67±0.035abce	1.61±0.047bce	1.88±0.044a	1.46±0.058e	1.53±0.031ce

68	2-Ethyl pyrazine	0.59±0.006d	0.73±0.058cd	0.74±0.052cd	0.72±0.035cd	0.91±0.023bc	1.26±0.058a	1.08±0.029ab	1.19±0.035a	1.09±0.052ab
69	2-Acetyl furan	0.82±0.01c	0.84±0.02c	1.35±0.101a	0.82±0.034c	0.79±0.01c	1.15±0.049b	0.93±0.045c	0.99±0.013bc	0.98±0.024bc
70	2-Pentyl furan	1.75±0.021ab	1.44±0.032c	1.19±0.051d	1.69±0.044ab	1.62±0.041bc	1.89±0.058a	1.67±0.056b	1.73±0.029ab	1.63±0.034bc
71	1-Octene	6.07±0.039de	5.7±0.106e	6.32±0.22cde	7.08±0.044bc	7.06±0.071bc	8.05±0.427a	6.77±0.033cd	7.08±0.066bc	7.8±0.176ab
72	2-Acetyl pyridine	0.62±0.012h	0.93±0.012g	1.22±0.072ef	1.06±0.029fg	1.41±0.061de	1.58±0.046cd	1.74±0.05c	2.27±0.041b	2.81±0.087a
73	4,5-Dimethylthiazole	0.77±0.003de	0.92±0.04cd	0.65±0.02e	1.01±0.092c	1.01±0.018c	0.98±0.032c	1.04±0.041c	1.32±0.01b	1.67±0.058a

Table S3. ROAV values of 28compounds during the 1st to 9th drying cycles

Compounds	ROAV								
	1st	2nd	3rd	4th	5th	6th	7th	8th	9th
2-Methyl butanoic acid methyl ester	100.0000	100.0000	100.0000	100.0000	100.0000	100.0000	100.0000	100.0000	100.0000
(R)-α-Pinene	0.0536	0.0256	0.0744	0.0292	0.0350	0.0435	0.0267	0.0363	0.0464
β-Myrcene	2.0623	1.3814	2.6201	1.1856	1.2024	3.5676	1.1687	2.3764	3.1210
(+)-Limonene	0.1074	0.1027	0.1886	0.0881	0.0929	0.1740	0.1120	0.1882	0.2408
2,3-Pentandione	1.9922	4.2467	1.4189	4.2728	5.8938	5.0713	6.3837	7.8796	8.6624
2-Butanone	0.0298	0.0347	0.0409	0.0420	0.0459	0.0458	0.0425	0.0458	0.0429
2-Butanone 3-hydroxy	0.0672	0.1426	0.1811	0.1043	0.1159	0.0883	0.1061	0.1431	0.1238
3-Mercapto-2-butanone	0.3798	0.7592	3.6681	0.6969	1.1190	0.9867	0.9108	1.2103	1.0140
Maltol D	0.0000	0.0001	0.0001	0.0001	0.0001	0.0001	0.0001	0.0003	0.0003
(E)-2-Heptenal D	0.3296	0.4613	0.6980	0.3860	0.3914	0.3974	0.4959	0.5420	0.4066
1-Hexanal	4.1712	5.6396	4.8472	6.6031	3.7500	6.0737	7.4578	6.1624	6.7070
2-Hexenal	0.0486	0.0815	0.1029	0.0683	0.0927	0.0782	0.0929	0.1248	0.1059

3-Methyl butanal D	46.1556	64.8649	56.8559	58.8247	88.1071	86.3587	70.0843	84.3542	92.2166
Butanal	7.3098	10.5209	27.0321	4.9627	5.2381	5.1648	5.2680	6.5759	5.7984
Heptanal	0.6001	0.5411	0.7216	0.4888	0.3489	0.2900	0.4006	0.3310	0.2932
n-Nonanal	0.8271	1.4363	1.3924	1.2018	1.6122	1.4405	1.8485	1.7649	1.3267
n-Octanal D	2.4775	2.7133	4.4942	2.3360	2.0504	1.7933	2.2920	1.9640	1.8928
3-Methyl butyl acetate	5.8534	11.1471	9.7351	10.8007	11.4603	10.8223	12.9659	16.6544	16.2314
(Z)-3-Hexenol	0.3017	0.4768	0.7739	0.3489	0.3590	0.3538	0.5301	0.7403	0.6036
1-Propanol, 2-methyl-	0.0024	0.0004	0.0203	0.0004	0.0004	0.0004	0.0003	0.0002	0.0003
2-Heptanol	0.0062	0.0173	0.0152	0.0205	0.0205	0.0123	0.0205	0.0135	0.0159
Methyl eugenol	0.0340	0.0283	0.0631	0.0371	0.0254	0.0276	0.0231	0.0270	0.0401
2-Methylbutanoic acid	0.0005	0.0008	0.0014	0.0009	0.0012	0.0011	0.0014	0.0014	0.0013
2-Methylpropionic acid	0.000015	0.000029	0.000063	0.000031	0.000038	0.000030	0.000038	0.000052	0.000055
Butanoic acid	0.0021	0.0039	0.0045	0.0026	0.0032	0.0028	0.0043	0.0075	0.0063
2, 3, 5-Trimethyl pyrazine	0.064520693	0.188697789	0.201984915	0.237300843	0.262337662	0.125441144	0.212924425	0.114324052	0.152866242
2-Acetyl furan	0.000382879	0.000605405	0.001414847	0.000507216	0.000564286	0.000678133	0.000672289	0.000876753	0.000749045

Table S4. Partial correlation coefficients between color parameters and chemical classes, controlling for steaming cycle number (n = 9)

Category	L*			a*			b*			E*ab		
	r	*p*	Bootstrap 95% CI	*r*	*p*	Bootstrap 95% CI	*r*	*p*	Bootstrap 95% CI	*r*	*p*	Bootstrap 95% CI
Terpenes	0.5	0.21	-0.544,0.933	-0.36	0.38	-0.944,0.780	0.361	0.38	-0.625,0.994	-0.428	0.29	-0.979,0.588
Ketones	-0.266	0.52	-0.946,0.833	0.267	0.52	-0.510,0.943	-0.155	0.71	-0.932,0.789	0.164	0.698	-0.835,0.936
Aldehydes	-0.009	0.98	-0.745,0.800	0.476	0.23	-0.258,0.935	-0.465	0.25	-0.954,0.188	0.464	0.247	-0.254,0.974
Esters	-0.313	0.45	-0.984,0.896	-0.177	0.68	-0.949,0.875	0.68	0.06	-0.239,0.970	-0.783	0.022	-0.975,-0.009
Alcohols	0.305	0.46	-0.672,0.955	-0.305	0.46	-0.893,0.774	0.386	0.35	-0.700,0.981	-0.521	0.186	-0.997,0.604
Acids	0.583	0.13	-0.922,0.925	-0.213	0.61	-0.878,0.886	-0.121	0.78	-0.877,0.823	0.312	0.452	-0.723,0.949
Pyrazines	-0.746	0.03	-0.975,-0.183	0.693	0.06	-0.137,0.976	-0.551	0.16	-0.982,0.252	0.432	0.285	-0.233,0.968
Furan	0.153	0.72	-0.771,0.838	0.318	0.44	-0.779,0.855	-0.225	0.59	-0.732,0.689	0.217	0.606	-0.831,0.78
Others	0.151	0.72	-0.643,0.934	-0.122	0.77	-0.924,0.930	0.288	0.49	-0.857,0.867	-0.383	0.349	-0.909,0.796

Note: Values are partial correlation coefficients (r) with 95% confidence intervals (percentile bootstrap, 1000 samples) in parentheses. Others: nitrogen-containing heterocyclic compounds (e.g., 2-acetylpyridine) and sulfur-containing compounds (e.g., 4,5-dihydro-3(2H)-thiophenone, 4,5-dimethylthiazole).
